# Novel RNA biomarkers improve discrimination of children with tuberculosis disease from those with non-TB pneumonia after *in vitro* stimulation

**DOI:** 10.3389/fimmu.2024.1401647

**Published:** 2024-09-26

**Authors:** Ortensia Vito, Stelios Psarras, Angeliki Syggelou, Victoria J. Wright, Virginia Amanatidou, Sandra M. Newton, Hannah Shailes, Katerina Trochoutsou, Maria Tsagaraki, Michael Levin, Myrsini Kaforou, Maria Tsolia

**Affiliations:** ^1^ Department of Infectious Disease, Faculty of Medicine, Imperial College London, London, United Kingdom; ^2^ Centre for Pediatrics and Child Health, Imperial College London, London, United Kingdom; ^3^ Center of Basic Research, Biomedical Research Foundation, Academy of Athens , Athens, Greece; ^4^ Second Department of Pediatrics, National and Kapodistrian University of Athens (NKUA), School of Medicine, P. and A. Kyriakou Children’s Hospital, Athens, Greece

**Keywords:** tuberculosis, transcriptomics, stimulant, PBMC, peripheral immunity signatures, biomarkers

## Abstract

The diagnosis of pediatric tuberculosis (TB) poses a challenge for clinical teams worldwide. TB-mediated changes in the expression of host genes in the peripheral blood can serve as diagnostic biomarkers and can provide better insights into the host immune mechanisms of childhood TB. Peripheral blood mononuclear cells (PBMCs) from children (n=102) with microbiologically confirmed TB disease, TB infection (TBI), pneumonia, and healthy controls (HC) were stimulated with either the Purified Protein Derivative (PPD) or the Early Secretory Antigen 6kDa-Culture Filtrate Protein 10 (ESAT6-CFP10) complex of *Mycobacterium tuberculosis* (*Mtb*). RNA was extracted and quantified using gene expression microarrays. Differential expression analysis was performed comparing microbiologically confirmed TB to the other diagnostic groups for the stimulated and unstimulated samples. Using variable selection, we identified sparse diagnostic gene signatures; one gene (*PID1*) was able to distinguish TB from pneumonia after ESAT6-CFP10 stimulation with an AUC of 100% in the test set, while a combination of two genes (*STAT1* and *IFI44*) achieved an AUC of 91.7% (CI_95%_ 75.0%-100%) in the test set after PPD stimulation. The number of significantly differentially expressed (SDE) genes was higher when contrasting TB to pneumonia or HC in stimulated samples, compared to unstimulated ones, leading to a larger pool of candidate diagnostic biomarkers. Our approach provides enlightened aspects of peripheral TB-specific responses and can form the basis for a point of care test meeting the World Health Organization (WHO) Target Product Profile (TPP) for pediatric TB.

## Introduction

Tuberculosis (TB), with 10.6 million new cases and 1.6 million deaths in 2021 (1) is still one of the major causes of mortality and morbidity worldwide with about 11% of the annual cases and 16% of all deaths occurring in children under 15 years of age (1, 2). TB is an airborne infectious disease caused by *Mycobacterium tuberculosis (Mtb)*, a primarily pulmonary pathogen that, occasionally, can spread to other tissues causing extra-pulmonary disease. Most of the current TB diagnostic tests are based on detecting the pathogen in sputum or biological samples from the site of disease by smear microscopy, microbiological culture, and polymerase chain reaction (PCR). However, in addition to the limitations of the current diagnostics, collecting biological samples from pediatric patients is a challenging procedure (3, 4). Notably, the lack of rapid, accurate and inexpensive diagnostic tests is one of the reasons why over 40% of the TB cases remain unreported or undiagnosed (1). This is a critical obstacle for the World Health Organization (WHO) End TB Strategy to reduce, by 2035, TB incidence and mortality by 90% and 95%, respectively, compared to the 2015 baselines (5). New tools are required to achieve these targets, especially in children where TB diagnosis is even more difficult. Importantly, radiographic findings are non-specific and could be consistent with other conditions including pneumonia. The tuberculin skin test (TST) and interferon gamma release assay (IGRA) cannot differentiate TB disease from infection, while diagnoses made on clinical grounds suffer from overlapping signs and symptoms with common pediatric diseases. On the other hand, the detection of *Mtb* in sputum can provide false negative results (6) and it may require more invasive sampling (i.e., induced sputum, gastric aspiration) in a pediatric population since young children cannot spontaneously expectorate sputum. Smear microscopy, *Mtb* culture, and even recently introduced point-of-care molecular tests (i.e., Xpert MTB/RIF Ultra assays) are microbiological tests of rather low sensitivity (7, 8). Therefore, there is an urgent yet unmet need for new, non-sputum, rapid, and non-invasive diagnostic tests for childhood TB. Their characteristics should comply with the high-priority target product profiles (TPP) for TB diagnostics as specified by WHO (9).

Recent evidence suggests that blood-based gene expression signatures for TB can accurately distinguish between microbiologically confirmed TB and TB infection (TBI), other respiratory infections or healthy controls (HC) (10–13), predict the progression from TBI to TB disease (14, 15) or monitor response to treatment (12, 16, 17). In particular, a 3-gene signature was identified by Sweeney et al. (12) through the meta-analysis of 14 data sets of studies mostly conducted in adults and can discriminate TB from other diseases and from TBI. The signature has been incorporated in the GeneXpert platform and the test is named Xpert MTB Host response (18).

Although a lot of studies have discovered TB signatures in adults, only two studies to date share data on TB diagnostics *vs.* other pulmonary diseases in children and both present a clear pattern of hypothesis-free, wide transcriptome analysis. Verhagen and colleagues published in 2013 the first microarray profiling study for pediatric TB biomarker identification in Warao Amerindian children (19). A signature of 116 genes identified by the random forest algorithm successfully distinguished TB cases from TBI individuals and HC in the training set. The signature was subsequently validated in publicly available adult datasets by reverse transcription quantitative real-time PCR (RT-qPCR). In 2014, Anderson and colleagues (11) described the discovery of transcriptional signatures for distinguishing microbiologically confirmed TB from other diseases with symptoms and clinical findings suggestive of TB, in a South African and Malawian pediatric population, comprising HIV-infected and HIV-uninfected children. Microarray analysis of whole blood was performed. Elastic net identified a 51-transcript signature with a sensitivity of 82.9% (CI_95%_ 68.6%–94.3%), and a specificity of 83.6% (CI_95%_ 74.6%–92.7%) when validated in an independent cohort recruited in Kenya. In 2016 Zhou and colleagues (20) identified an 8-miRNA signature with 95.8% sensitivity and 100% specificity for the discrimination of TB *vs.* uninfected healthy controls. In a recent study (21), the three-gene signature discovered by Sweeny et al. (12) was tested in children with TB from a number of African countries and India. The sensitivity of the test in culture confirmed TB was found at 59.8% (CI_95_%:50.8-60.4) and the specificity 90.3% (CI_95_%:85.5-94.0).

In addition to whole blood, transcriptomic profiles of peripheral blood mononuclear cells (PBMCs) have also been used to discriminate between TB and other disease states (22, 23). In 2013, Dhanasekaran and colleagues (24) identified a 5-gene signature that can discriminate TB from HC and an 11-transcript signature that can discriminate TB from TBI, in a pediatric population in India. However, such approaches partially neglect TB-specific immunological responses, omitting the exposure of the biological samples to *Mtb*-derived antigens, a practice commonly used for decades in TB diagnostic tests. In particular, the monitoring of the response to purified protein derivate (PPD), which is a mixture of *Mtb*-derived antigens, has been used as an *in vivo* skin test to identify TB infection or disease. Accordingly, gene expression analysis following PBMC exposure to PPD has been employed (25, 26) to identify biomarkers discriminating TB from TBI. On the other hand, the early secretory antigen 6kDa (ESAT-6) and the culture filtrate protein 10 (CFP-10) antigens are the basis of the IGRA test, a major *in vitro* diagnostic tool for TB (27), eliciting responses comparable to that of PPD in pediatric PBMCs (28). ESAT-6 and CFP-10, as well as additional *Mtb* proteins, have been applied to elicit and monitor gene expression changes in PBMCs (29, 30). However, biomarker discovery studies combining or comparing responses to different *Mtb* antigen formulations are currently lacking.

Most importantly, the majority of whole blood profile-based studies have been focusing on adult populations with signatures underperforming when applied to pediatric cohorts. The few relevant pediatric studies are characterized by overrepresentation of specific populations, mainly from African or Asian countries (16, 31–33). These studies identified large signatures which may not be suitable for point of care testing.

In the current study, we have investigated the *Mtb*-specific effect on the transcriptomic profiles of PBMCs isolated from children with microbiologically confirmed TB, TBI, pneumonia or HC using *in vitro* stimulation with PPD or ESAT6-CFP10 complex. To our knowledge, this is the first PBMC-based expression profile study exploring pediatric immune responses to *Mtb* before and after *Mtb* antigen stimulation. We hypothesized that *ex vivo* stimulation would amplify differences in TB specific host response genes providing stronger disease biomarkers. The results obtained may help define a new set of candidate transcriptomic biomarkers to diagnose TB in children after blood stimulation and provide a more complete picture of the host immune response to *Mtb*.

## Materials and methods

### Patients

Children under 16 years of age with TB disease, TBI or community-acquired pneumonia as well as HC were prospectively recruited at the Second Department of Paediatrics of the National Kapodistrian University Athens (NKUA) at the P. and A. Kyriakou Children’s Hospital over 5 years. Subjects with symptoms compatible with TB or a history of contact with adult infectious cases and/or a positive TST were evaluated for TB disease or TBI.

Patients were diagnosed with TBI when they had both QuantiFERON-TB Gold In-Tube test (QFT-GIT) and TST positive, no symptoms or clinical findings, and a normal chest X-ray. All cases with TB disease were confirmed by culture and/or Xpert Mtb/RIF. Pneumonia cases were diagnosed based on clinical and radiological findings (alveolar infiltrates) and a low index of suspicion for TB, whereas HC were asymptomatic children admitted for routine surgical procedures (i.e., tonsillectomy). In both pneumonia and HC, TST was negative prior to recruitment. The complete diagnostic algorithm is shown in **Supplementary Figure 1**.

The standard dose of Tuberculin PPD RT 23 SSI (2 TU), manufactured by the Statens Serum Institut (SSI) in Copenhagen (Denmark), was used for the TST. The QFT-GIT (Cellestis Limited, Carnegie, Victoria, Australia) was performed per manufacturer’s instructions at our research laboratory as a blind experiment (i.e., it was unknown which disease group each sample belonged to). TSTs with an induration > 5 mm were considered positive. Children with indeterminate QFT-GIT results were excluded from the study. Blood samples for transcriptomic analysis were collected from all participants.

The subjects were enrolled in two distinct time frames and were accordingly grouped in two different sets with similar clinical characteristics. Participants enrolment took place from 10/09/2010 until 28/05/2012 and from 12/07/2012 until 17/05/2016. Data from the two sets were merged to identify the SDE genes between TB and the other clinical groups and to study the host immune mechanisms of childhood TB. For signature identification the samples were split in a balanced way into a training set (70% of the data) and a test set (the remaining 30% of the data) (**Supplementary Figure 2**). Differential expression analysis and signature discovery were run on the training set, and the findings were then evaluated on the test set.

All data including demographics, socioeconomic factors, previous medical history, presence of symptoms, details about the index case and degree of exposure, history of BCG immunization and presence of a scar, clinical findings, TST results, chest X-ray findings, QFT-GIT result, culture, and transcriptomic analysis were collected into a database.

### Setting

The TB clinic at the P. and A. Kyriakou Children’s Hospital is a reference center for pediatric TB for the greater Athens area, which has a population of about 3.8 million people according to the most recent estimate of the Hellenic Statistical Authority (https://www.statistics.gr/en/home). Pediatric TB cases were also referred from other central and southern regions of the country. Children were referred for evaluation if there was close contact with an adult infectious TB case or a positive TST during screening. On rare occasions, children were referred for evaluation because of the presence of symptoms or clinical findings suggestive of TB.

### Sample collection, RNA extraction and processing

**Figure 1:**
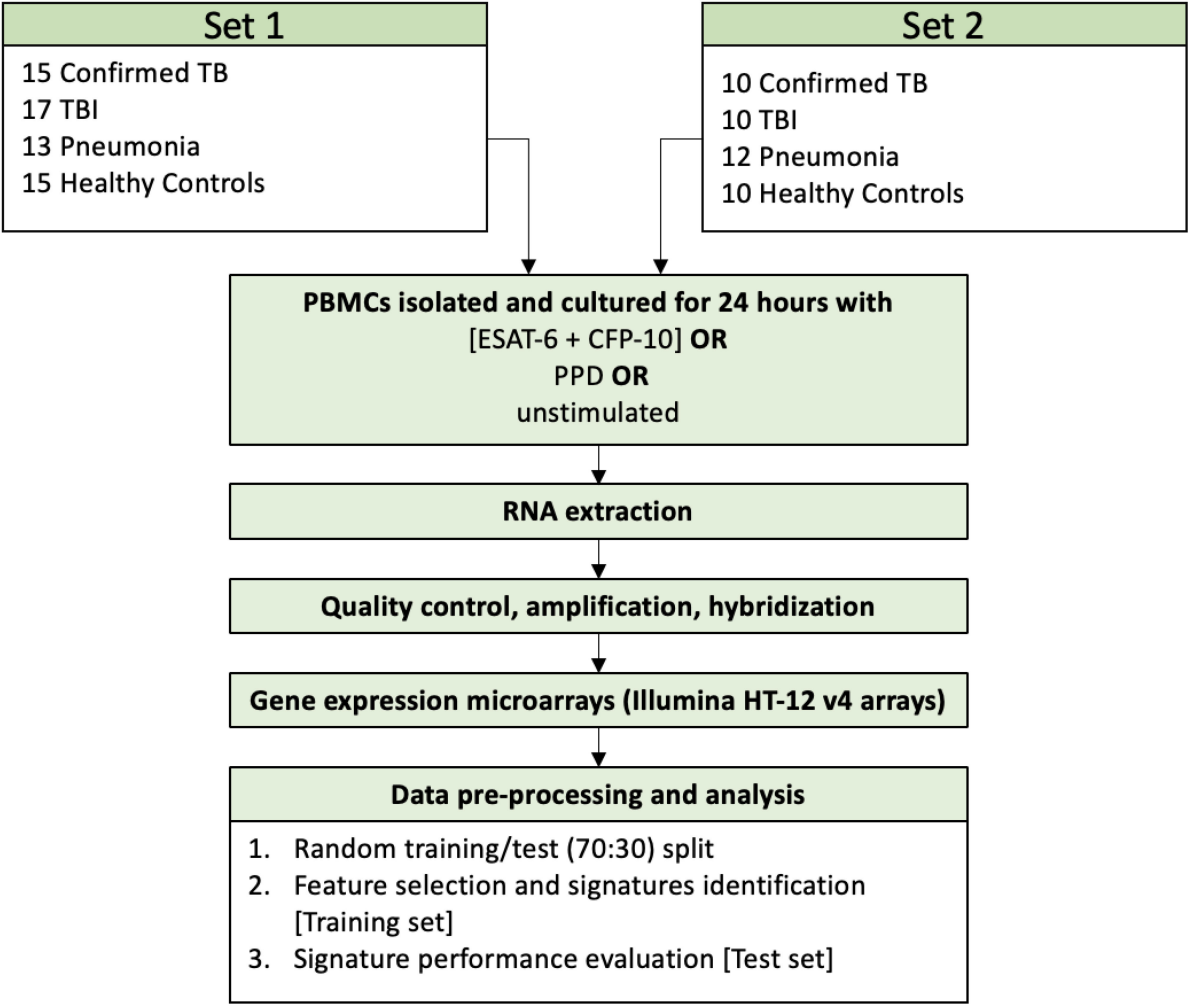
Schematic overview of sample collection, treatment, and analysis in this study.

From each study subject, 5 ml of whole peripheral blood was collected in heparinized tubes. In the patient groups the blood samples were collected before treatment for TB disease or TBI was initiated. PBMCs were isolated according to the Histopaque 1077 (Sigma-Aldrich) cell isolation procedure, with a mean recovery of 6x10^6^ cells per sample. The isolated PBMCs were cultured in 0.2 ml growing medium (RPMI, 10% serum & antibiotics) and were exposed for 24 hours in triplicate to a) recombinant *Mtb* antigens, ESAT-6 and CFP-10 (LIONEX Diagnostics and Therapeutics, Braunschweig, Germany), 10 μg/ml each, b) Tuberculin PPD (for *in vitro* tests, Statens Serum Institut, Copenhagen, Denmark), 1 mg/ml, or c) remained in the nutrient agent without stimulation. 150μl of supernatant was removed from each well and the remaining culture medium (50 μl) was triturated to resuspend the attached cells, transferred to a new vial, and centrifuged. The supernatant was discarded and 50 μl of Trizol reagent (Invitrogen Life Technologies) was added to the cell pellet. After washing the cell monolayer with PBS, another 50μl of Trizol reagent was added to the culture plate, incubated for 5 minutes at RT, before being added to the cell pellet. The combined 100 μl Trizol cell lysate was stored at -80°C. Total RNA was isolated from pooled replicates for each condition per patient using a modified RNeasy protocol (Qiagen). After QC200 ng of RNA was converted to biotinylated cRNA using Illumina TotalPrep RNA Amplification kits (Applied Biosystems) and 750ng of the labelled cRNA hybridized to Human HT-12 V4 Expression BeadChip arrays (Illumina) following manufacturer’s instructions. The overall procedure and sample sizes are depicted in **Figure 1**.

### Data and statistical analysis

Data analysis was conducted using the R programming language (version 4.1.2) (34). Background correction, variance stabilization and robust spline normalization (35) were performed on the raw intensities for the two independent sets separately using the *lumi* (36) package. Expression values were transformed to a logarithmic scale (base 2). Principal Component Analysis (PCA) was used for quality control of the normalized data. The outliers, 7 in set 1 and 2 in set 2, were excluded and the array effect in the second set was corrected using the removeBatchEffect function in the *limma* package (37). Visual inspection of the PCA plot confirmed the correction of the array effect (**Supplementary Figure 3**).

In order to explore the host immune mechanisms of childhood TB captured by the data, the two sets were merged, and all samples used to run the differential gene expression analysis using the *limma* package (37) to identify the set of Significantly Differently Expressed (SDE) genes subsequently subjected to pathway analysis. The ComBat function in the *sva* package (38) was used to correct the batch effect prior to differential expression analysis (**Supplementary Figure 4**). TB was contrasted to TBI, HC and pneumonia for each stimulant (PPD and ESAT6-CFP10 complex), as well as for the unstimulated samples accounting for age and sex at birth. Adjustment for false discovery rate was performed using the Benjamini-Hochberg correction (39). Genes with an adjusted p-value less than 0.05 and an absolute value of log2 Fold-Change (LFC) over 1 were considered SDE. The lists of SDE genes were subjected to functional enrichment analysis using the *gprofiler2* package (40) to determine which pathways were significantly upregulated or downregulated in each disease group comparison, for both stimulated and unstimulated samples. Functional enrichment analysis was run by making use of the pathway data in the Kyoto Encyclopedia of Genes and Genomes (KEGG) (40).

FS-PLS (Forward Selection-Partial Least Square), an iterative feature selection algorithm that selects a minimum set of features with high predictive accuracy excluding the correlated variables (13, 41, 42), was then run on the training set to identify novel and unique small gene expression signatures for differentiating TB from the other disease groups. The data split into training and test sets (70% - 30%) was done using the R set.seed() function (base package) and a custom function taking into account the total number of samples in each set and in each group (unstimulated, ESAT6-CFP10 and PPD stimulated samples) to create balanced subsets (**Supplementary Figure 2**). The selected features were then weighted using a logistic regression model. The final signatures were tested on the test set and performance, in terms of area under the Receiver Operating Characteristic (ROC) curve (AUC), sensitivity and specificity, was assessed for both training and test sets.

## Results

### Patient cohort

Overall, 102 children were enrolled in the study and were classified into 4 different groups, microbiologically confirmed TB disease (n=25), TBI (n=27), pneumonia (n=25) and HC (n=25). Median age was 5.6 years (Interquartile Range 1.4-12.4) and females represented half of the samples (50%). Although most participants were born in Greece (82%), it was estimated that 37% of the enrolled subjects were children of immigrant families (CIF). All but 4 children with TB disease were TST positive while the QFT-GIT test was positive in all but one of these patients. Both TST and QFT-GIT were positive in all TBI cases. HIV serology was negative in all participants.

Clinical, radiological and laboratory characteristics of patients are described in **Supplementary Tables 1** and **2**.

### Transcriptomic profiles of PBMCs upon stimulation

#### TB *vs*. Pneumonia

**Figure 2:**
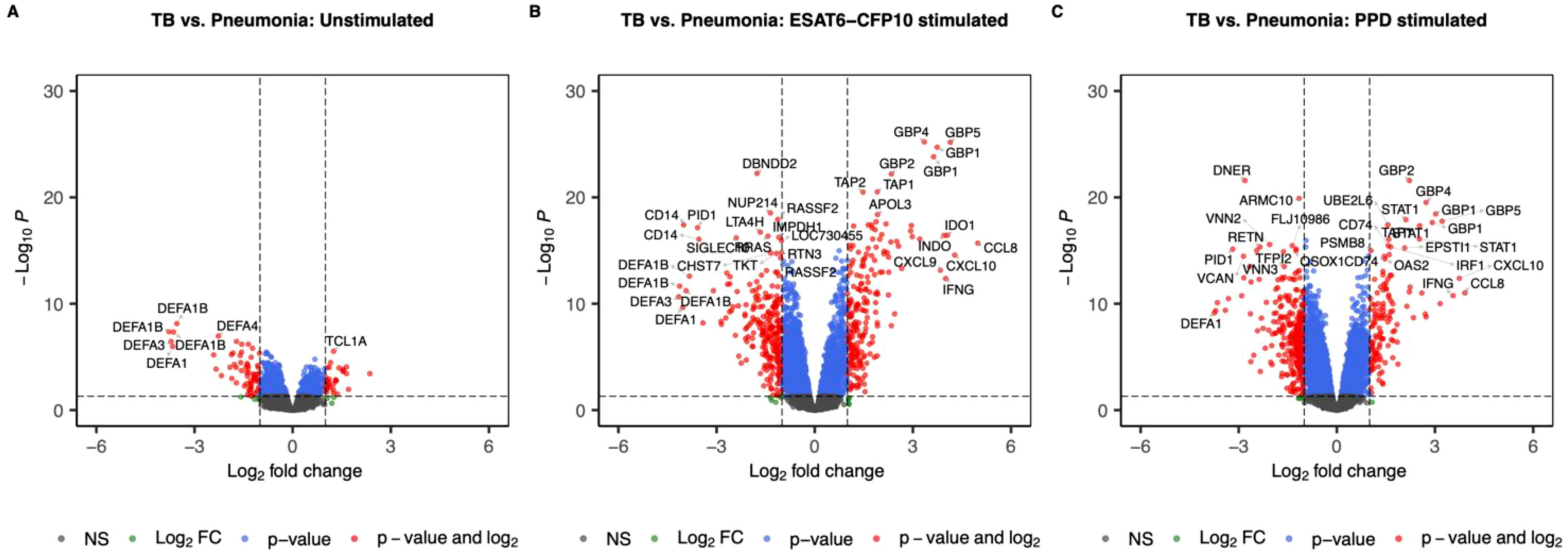
Volcano plots showing log2 Fold-Change (LFC) and -log10 adjusted p-values from differential expression analysis comparing TB *vs.* Pneumonia in the unstimulated samples **(A)**, in the ESAT6-CFP10 stimulated samples **(B)** and in the PPD stimulated samples **(C)**. Red dots represent the genes with adjusted p-value < 0.05 and absolute LFC > 1; blue dots represent the genes with adjusted p-values < 0.05 and absolute LFC < 1; green dots represent the genes with adjusted p-values > 0.05 and absolute LFC > 1; black dots represent the non-significant genes (adjusted p-values > 0.05 and absolute LFC < 1).

First, we set out to identify the effect of ESAT6-CFP10 and PPD on the transcriptomic profiles of PBMCs in the different patient groups. A linear model accounting for age, sex and experimental site was used on the whole cohort to identify the SDE genes when contrasting the disease groups in both unstimulated and stimulated samples. We identified 96 SDE genes (63 under- and 33 over-expressed in TB) when comparing TB *vs.* Pneumonia in the unstimulated samples (**Figure 2A**), 448 (239 under- and 209 over-expressed in TB) in the ESAT6-CFP10 stimulated sample (**Figure 2B**), and 430 (304 under- and 126 under-expressed in TB) in the PPD stimulated samples (**Figure 2C**).

#### TB *vs*. HC

**Figure 3:**
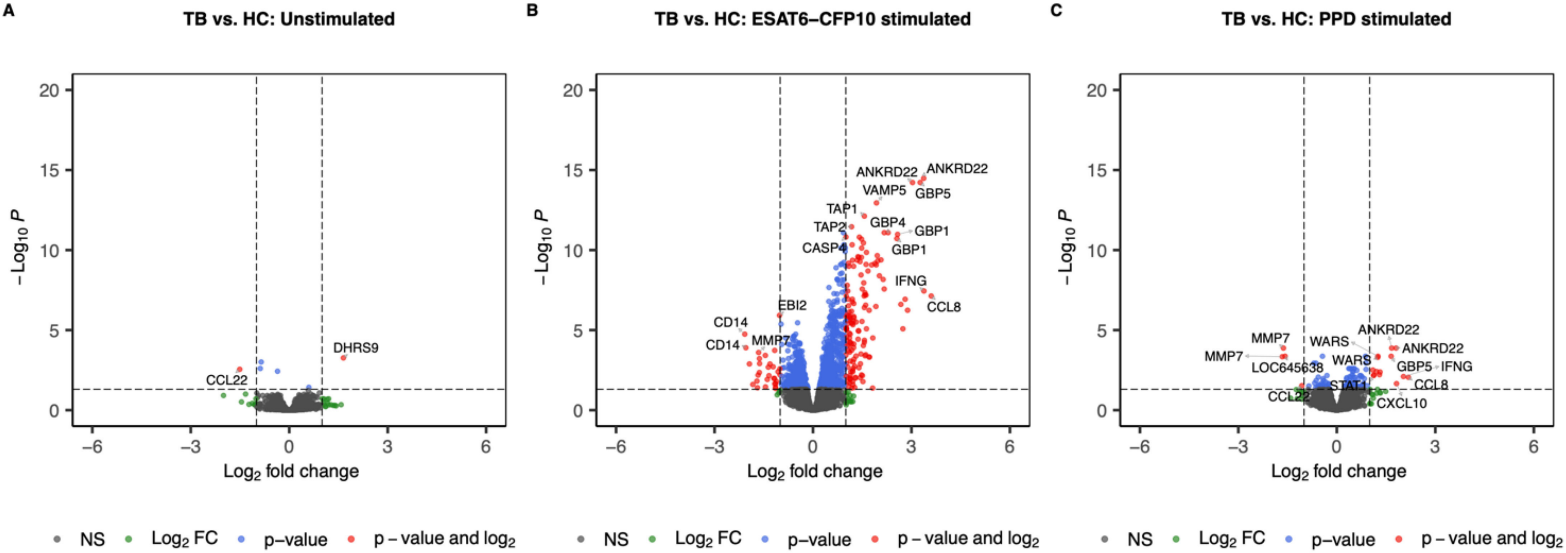
Volcano plots showing log2 Fold-Change (LFC) and -log10 adjusted p-values from differential expression analysis comparing TB *vs.* HC in the unstimulated samples **(A)**, in the ESAT6-CFP10 stimulated samples **(B)** and in the PPD stimulated samples **(C)**. Red dots represent the genes with adjusted p-value < 0.05 and absolute LFC > 1; blue dots represent the genes with adjusted p-values < 0.05 and absolute LFC < 1; green dots represent the genes with adjusted p-values > 0.05 and absolute LFC > 1; black dots represent the non-significant genes (adjusted p-values > 0.05 and absolute LFC < 1).

When contrasting TB *vs.* HC, we identified 2 SDE genes (1 under- and 1 over-expressed in TB) in the unstimulated samples (**Figure 3A**), 175 (30 under- and 145 over-expressed in TB) in the ESAT6-CFP10 stimulated sample (**Figure 3B**), and 18 (4 under- and 14 under-expressed in TB) in the PPD stimulated samples (**Figure 3C**).

#### TB *vs*. TBI

**Figure 4:**
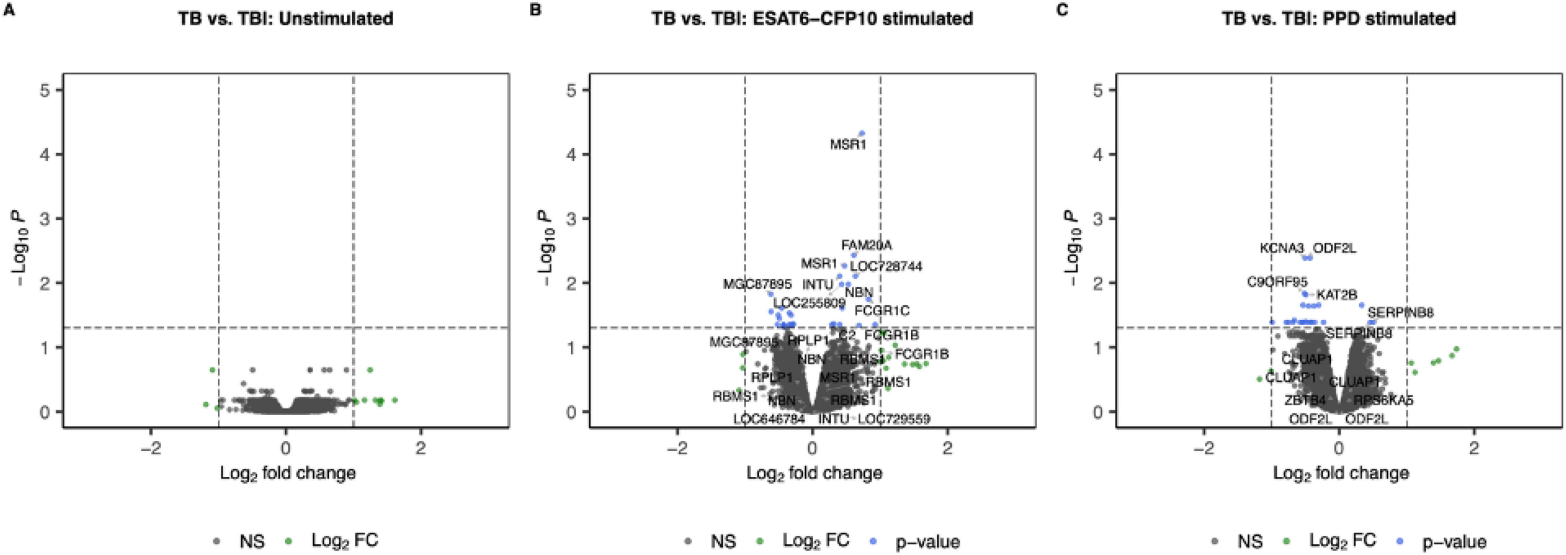
Volcano plots showing log2 Fold-Change (LFC) and -log10 adjusted p-values from differential expression analysis comparing TB *vs.* TBI in the unstimulated samples **(A)**, in the ESAT6-CFP10 stimulated samples **(B)** and in the PPD stimulated samples **(C)**. Red dots represent the genes with adjusted p-value < 0.05 and absolute LFC > 1; blue dots represent the genes with adjusted p-values < 0.05 and absolute LFC < 1; green dots represent the genes with adjusted p-values > 0.05 and absolute LFC > 1; black dots represent the non-significant genes (adjusted p-values > 0.05 and absolute LFC < 1).

When contrasting TB *vs.* TBI, no genes reached the thresholds for FC and adjusted p-value (**Figure 4**).

#### Comparison of stimulated vs. unstimulated samples

**Figure 5:**
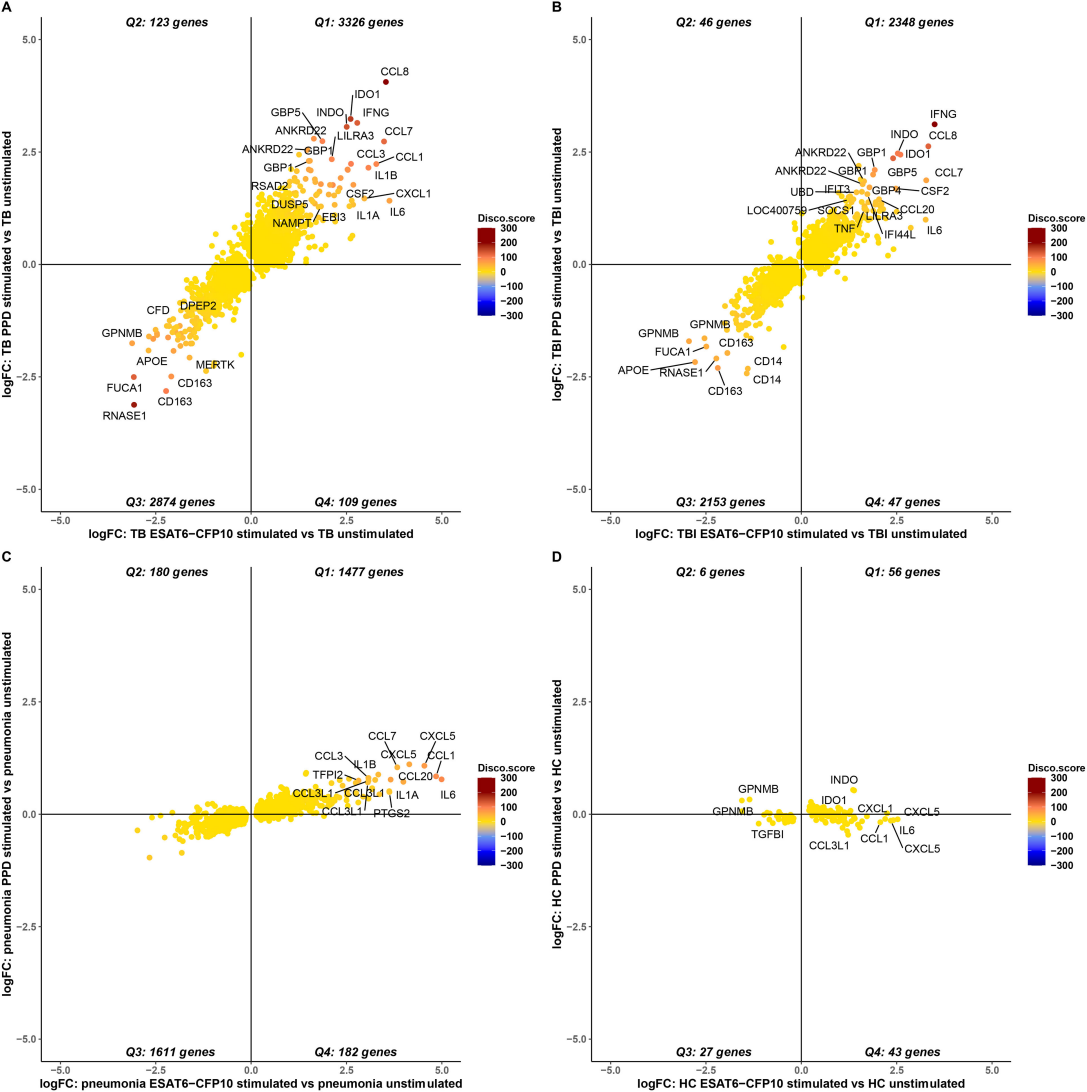
Concordance and discordance of the log2FoldChange (LFC) of significant genes (adjusted p-value <0.05) in TB **(A)**, TBI **(B)**, Pneumonia **(C)** and HC **(D)** comparing stimulated *vs.* unstimulated samples. The LFC of the significant genes for ESAT6-CFP10 stimulated *vs.* unstimulated samples is on the X axis, and the LFC of the SDE genes for PPD stimulated *vs.* unstimulated samples is on the Y axis. Each dot is colored according to the disco score and represents a gene: the stronger the red color the more concordantly regulated the gene pair; the stronger the blue color the more discordantly regulated the gene pair.

To evaluate the gene expression response to ESAT6-CFP10 and PPD stimulants, the stimulated samples were contrasted to the unstimulated samples for each disease group and the LFC of the significant genes (i.e., adjusted p-value <0.05) were compared (**Supplementary Figure 5**). **Figure 5** shows the concordant and discordant significant genes for TB (**Figure 5A**), TBI (**Figure 5B**), Pneumonia (**Figure 5C**) and HC (**Figure 5D**) when stimulated and unstimulated samples were compared. **Table 1** shows the number and percentage of significant genes that are concordant and discordant in each group. Genes encoding the inflammatory cytokines IL1 and IL6 and several members of the CCL and CXCL families of chemokines, as well as members of the interferon response pathways, were consistently over-expressed.

**Table 1:** Concordance and discordance of the log2FoldChange (LFC) in terms of number and percentage of significant genes (adjusted p-value <0.05) in TB, TBI, Pneumonia and HC comparing stimulated *vs.* unstimulated samples.

	Concordant	Discordant
**TB**	6200 (96.29%)	232 (3.61%)
**TBI**	4501 (97.98%)	93 (2.02%)
**Pneumonia**	3088 (89.51%)	362 (10.49%)
**HC**	83 (62.88%)	49 (37.12%)

#### Pathway analysis

**Figure 6:**
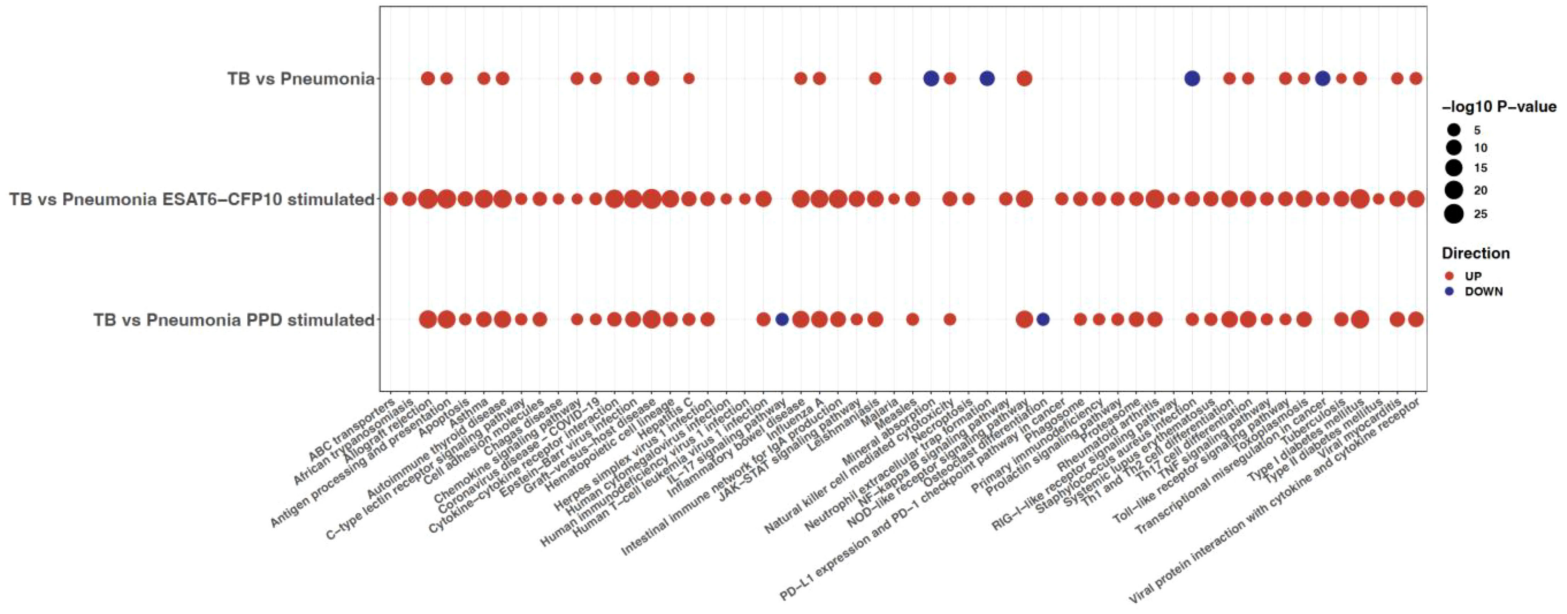
KEGG pathway analysis run on the SDE genes (adjusted p-value <0.05 and absolute LFC >1) from differential expression analysis comparing TB *vs.* Pneumonia for unstimulated and stimulated samples. The size of the dots is inversely proportional to the adjusted p-values, and the color represents the direction (up is red and down is blue). Only the pathways (columns) with a p-value <0.05 are shown.

A functional enrichment analysis querying the KEGG database was then run on the SDE genes (i.e., absolute value of LFC >1 and adjusted p-value <0.05) for each disease group comparison. **Figure 6** represents all the pathways (columns) which were significant when TB was contrasted to pneumonia for the unstimulated and stimulated samples (rows). This analysis highlighted that the number of significant pathways was much higher in the stimulated samples, with 52 pathways for the ESAT6-CFP10 stimulated samples and 42 for the PPD stimulated samples, compared to the 26 pathways of the unstimulated samples. The *NOD-like receptor signaling*, a major system primarily appointed in initiating the innate immune response and identifying pathogens (43), was upregulated when we compared TB *vs.* Pneumonia both in unstimulated and stimulated samples. Other innate immunity involving pathways, like *Natural killer cell mediated cytotoxicity* and *Toll-like receptor signaling pathway*, as well as for adaptive immunity-specific pathways, such as antigen presentation machinery and lymphocyte differentiation related signaling, including Th1, Th2 and Th17 cell differentiation, were also upregulated when we compared TB *vs.* Pneumonia. Some innate immunity involving pathways, like the one of C-type lectins (44), were preferentially activated in the stimulated samples. This was also true for pathways involved in interferon response, such as the JAK/STAT (45), and in cytokine and chemokine signaling, including TNFα. Notably, pathways mediating immune response to cytosolic DNA and RNA sensing (46), including the RIG-I pathway (47), were up-regulated in ESAT6/CFP10- but not in PPD-stimulated TB samples (**Figure 6**). In contrast, we noticed down-regulation of the proinflammatory interleukin-17 (IL-17) pathway in PPD-stimulated TB samples and of innate immunity involved NETosis-pathways (48) in unstimulated TB. The *Tuberculosis* pathways was significantly upregulated in both unstimulated and stimulated samples, while a defective mineral absorption status was revealed when comparing TB to pneumonia at their baselines. The significant pathways for the other disease group comparisons are shown in **Supplementary Figure 6**. Interestingly, when directly compared to PPD stimulation, ESAT/CFP10 stimulation appeared to be inferior in all groups in stimulating NOD-like pathways and aspects of cytokine signaling including NFkB, TNFα and IL17 pathways, further pointing out the necessity of multiple stimulation regimes to globally address TB responses in such approaches.

### Identification of diagnostic biomarkers for TB

In clinical practice, TB needs to be distinguished from diseases with similar clinical features and patient presentation therefore we sought to identify parsimonious gene expression signatures with diagnostic potential to distinguish TB from pneumonia. To that end, the training and the test datasets were randomly generated. The clinical characteristics are presented in **Supplementary Table 3**. Number, median age in years, interquartile range (IQR), and percentage of males in the microbiologically confirmed TB and pneumonia groups for the training and test sets are shown in **Table 2**.

**Table 2:** Number, median age in years, interquartile range (IQR), and percentage of males in the microbiologically confirmed TB and pneumonia groups for the training and test sets.

	Disease	Stimulant	Number	Age (years) median IQR	Sex (male, %)
**TRAINING SET**	Confirmed TB	NONE	12	3.2 (2.5-10.1)	50
		ESAT6- CFP10	13	2.0 (1.1-10.8)	69
		PPD	16	2.0 (1.1-5.3)	56
	Pneumonia	NONE	16	4.2 (1.8-6.1)	50
		ESAT6- CFP10	16	6.0 (5.1-8.2)	38
		PPD	17	4.8 (1.6-7.0)	29
**TEST** **SET**	Confirmed TB	NONE	7	2.0 (1.9-7.9)	100
		ESAT6- CFP10	8	3.1 (1.8-6.1)	62
		PPD	9	3.0 (1.8-5.0)	67
	Pneumonia	NONE	8	5.8 (4.0-6.5)	25
		ESAT6- CFP10	8	1.7 (1.5-3.8)	50
		PPD	8	5.6 (3.9-5.8)	62

A linear model accounting for age and sex at birth was used on the training set (70%) to calculate the adjusted p-value and LFC for each transcript when comparing microbiologically confirmed TB *vs.* pneumonia in both unstimulated and stimulated samples. We used FS-PLS on the SDE genes of the training set for each comparison, and then we assessed the performance of the signatures on the test set.

**Figure 7:**
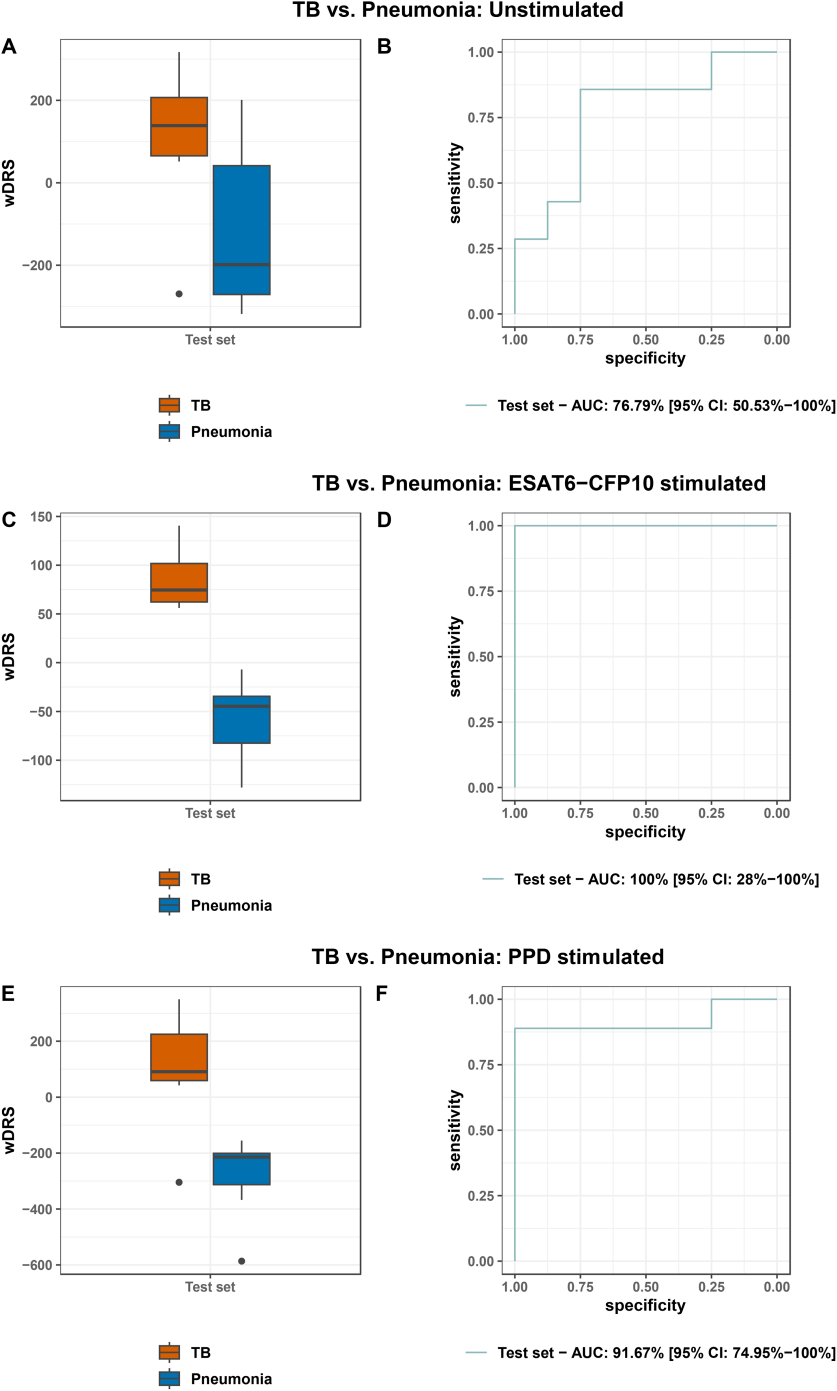
Boxplots and ROC curves of the weighted disease risk score of the signatures in the test set to distinguish TB from Pneumonia in unstimulated samples **(A, B)**, ESAT6-CFP10 stimulated samples **(C, D)** and PPD stimulated samples **(E, F)**.

FS-PLS selected a 2-gene signature (*ADA* and *HIST2H2AA3*) in the training set for distinguishing TB from Pneumonia in unstimulated samples. The genes combined in a weighted Disease Risk Score (wDRS) achieved an AUC of 76.8% (CI_95%_ 50.5%-100.0%) in the test set, with sensitivity of 85.7% (CI_95%_ 57.1%-100%) and specificity of 75.0% (CI_95%_ 35.7%-100.0%) (**Figures 7A, B**). FS-PLS selected a 1-gene signature (*PID1*) in the training set for distinguishing TB *vs.* Pneumonia in ESAT6-CFP10 stimulated samples. *PID1* achieved an AUC of 100% (CI_95%_: 28.0%-100.0%) in the test set, with sensitivity of 100.0% and specificity of 100% (**Figures 7C, D**). FS-PLS selected a 2-gene signature (*STAT1* and *IFI44*) in the training set for distinguishing TB *vs.* Pneumonia in PPD stimulated samples. The genes combined in a wDRS achieved an AUC of 91.7% (CI_95%_ 75.0%-100%) in the test set, with sensitivity of 88.9% (CI_95%_ 66.7%-100%) and specificity of 100.0% (**Figures 7E, F**). The performance of the signatures in the training and test sets are shown in **Supplementary Figure 7** and **Supplementary Table 4**. We have also assessed the performance of the signatures discovered in each stimulus-specific comparison on the rest of the samples (**Supplementary Table 4**). The signatures had the highest AUC in the test sets for the ESAT6-CFP10 stimulated samples, irrespective of the stimulus discovery set used. In addition, we assessed the performance of the 3-gene whole-blood Sweeney signature [12] in our dataset (**Supplementary Table 5**). Although discovered in unstimulated whole blood RNA samples, the 3-gene signature had the highest performance in the ESAT6-CFP10 stimulated PBMCs (training set: 89.9% (CI_95%_ 78.3%-100%) and test set: 89.06% (CI_95%_ 67.41%-100%)), with poor performance in the unstimulated PBMCs (training set: 49.5% (CI_95%_ 25.2%-73.7%) and test set: 64.29% (CI_95%_ 32.1%-96.47%)).

## Discussion

In this prospective study, transcriptomic profiles of PBMCs from children with TB, TBI, pneumonia and HC with and without stimulation with *Mtb* specific antigens were analyzed. We demonstrated that the number of SDE genes increased upon *in vitro* stimulation when TB was compared to pneumonia, as well as TBI and HC, with the effect being more prominent for ESAT6-CFP10 stimulation for the TB *vs.* pneumonia and HC comparisons. As a result, stimulation revealed a larger number of differentially affected signaling pathways underpinning pediatric TB. We also compared the PBMC response between PPD and ESAT-CFP10 stimulation in the different diagnostic groups highlighting the different genes and pathways involved in response to the two stimulants. Moreover, we showed that very sparse gene sets can effectively discriminate TB from pneumonia in both stimulated and unstimulated samples, with classification accuracy increasing in the stimulated samples. In terms of the WHO defined TPP, the sparse signatures identified in the stimulated samples achieved the WHO targets for sensitivity and specificity for a non-sputum based diagnostic test for pediatric TB (i.e., sensitivity >66% and specificity >98%) (9). A single gene achieved 100% sensitivity and specificity upon ESAT6-CFP10 stimulation, whereas, upon PPD stimulation, a 2-gene signature achieved a sensitivity of 88.9% (CI_95%_ 66.7%-100%) in the test set when the specificity was fixed at 98%.

The stimulation of PBMCs from TB patients, but also those with TBI, revealed a substantial increase of immune response-related pathways compared to unstimulated samples. On the other hand, the gene expression profiles of PBMCs from patients with pneumonia as well as those from HC were not perturbed as much in comparison to matched unstimulated samples. When we compared the effect of the two different stimulants used in the different diagnostic groups, the SDE genes were largely concordant between the two stimulants in terms of direction and significance for the TB and TBI groups, which is expected given the specificity of these stimulants. The number of SDE genes for pneumonia and HCs upon stimulation was lower, and with lower degree of concordance. This can be explained by small non-specific effects of the stimulation in these groups, while the lack of concordance by statistically false positive results. In terms of the comparisons between different disease groups, for the TB *vs*. HC comparison, more genes were SDE upon ESAT6/CFP10 stimulation as compared to PPD stimulation (175 *vs.* 18). In addition, ESAT6/CFP10 unveiled a larger number of significant pathways in TB when compared to HC samples than PPD (58 *vs*. 23). Similar trends were found in the TB *vs*. pneumonia comparison. Since, the ESAT6 and CFP10 proteins are encoded by the RD1 genes *Rv3874* and *Rv3875* of *M. tuberculosis* which are absent from all strains of Bacillus Calmette-Guérin (BCG) and from most strains of nontuberculous mycobacteria, it is expected for them to elicit more specific responses compared to PPD which contains a mixture of proteins shared by *Mtb*, BCG and nontuberculous mycobacteria. The ESAT6 and CFP10 proteins have been used in the interferon-gamma release assays (IGRAs) which have been shown to have higher specificity in comparison to tuberculin skin test (TST) where PPD is used. In this study, it was shown that the combination of these two peptides can also induce much bigger changes in gene expression profiling in comparison to PPD.

In the TB *vs.* pneumonia comparison, out of the 96 SDE genes in the unstimulated samples, 81 and 82 were also SDE after stimulation with ESAT6-CFP10 and PPD respectively, while 542 new genes were found to be SDE after stimulation with the two stimulants, with 173 in common (**Supplementary Figure 8**). **Supplementary Tables 6**–**8** list the SDE genes when contrasting TB *vs.* Pneumonia for unstimulated, ESAT6-CFP10 stimulated and PPD stimulated samples, respectively. Previously identified key genes involved in the host response in patients with pediatric TB were identified as SDE upon stimulation. These include members of the guanylate-binding proteins family (i.e., *GBP1*, *GBP2*, *GBP4*, *GBP5*), which play a central role in the immune response to infection and inflammation (49) and have been previously reported as TB biomarkers (12, 50–53). Moreover, *STAT1*, involved in the immune defense during TB disease (54), was found to be over-expressed in TB in both unstimulated and stimulated samples, along with the chemokine-encoding genes *CXCL9* and *CXCL10*. In addition, the RNA levels of three α-defensins (*DEFA1*, *A1B*, and *A3*) important for host anti-microbial and anti-TB defense (55, 56), were lower in the TB groups, suggesting a TB-specific impairment of the hosts’ immune status as compared to pneumonia. *CD14*, a marker of human monocytes known to be critically involved in *Mtb* infection and progression to TB disease (57, 58), was also under-expressed. Notably, *PID1* (Phosphotyrosine Interaction Domain Containing 1, also known as *NYGGF4*), which encodes a molecular adaptor important for glucose homeostasis (59) and is expressed by human monocytes in a IFN-γ-dependent manner (60), was under-expressed in the PMBCs from TB patients upon stimulation with both stimulants in comparison to pneumonia.

Both adaptive and innate immunity related pathways were found to be differentially activated or suppressed in the TB *vs.* pneumonia comparison and the other groups as well (**Figure 6**; **Supplementary Figure 6**). Two pathways of macrophage deaths, apoptosis and necroptosis, that play a role in host antimicrobial defense in the early TB infection (61), were found to be significant upon sample stimulation in TB *vs.* pneumonia. The “JAK-STAT signaling pathway” and “RIG-I-like receptor signaling pathway”, which also become significant after stimulation, have been shown previously to be involved in type I IFN detrimental effect in TB disease (62) and in regulation of type I IFN abundance (63) respectively. Other pathways, such as “C-type lectin receptor”, “Cytokine-Cytokine receptor interaction”, “NF-kappa B signaling pathway”, and “TNF signaling pathway”, that become significant upon sample stimulation, suggest a stronger innate and adaptive host immune response compared to the unstimulated samples. Features of impaired adaptive immunity including defective Th1 and cytotoxic responses (produced by CD4^+^ and CD8^+^ cells, respectively), as well as hyperresponsive Th17 lymphocytes are known to underlie TB disease (64). Previous transcriptomic analyses highlighted those acquired immune defects in pediatric TB (65). Accordingly, activated Th17 cell differentiation was significant in TB as compared to pneumonia, with lower p value upon stimulation.

These pathways also conform well to several of the highly changed SDE genes (**Figure 2**). For instance, under-expression of *CD14* and *MTI1H* (encoding metalothionin 1, a promoter of circulating monocyte survival) in TB *vs.* pneumonia may mirror a compromised monocyte/macrophage potential in TB (66). However, molecules either enhancing human macrophage anti-TB activity (*IFI44L*) (67), or suppressing RIG-I mediated innate immunity (*UBD*, also known as *FAT10*) were simultaneously found to be overexpressed in the TB group (68). On the other hand, both over-expression of *INDO*/*IDO1* (69) and under-expression of *SIGLEC10* may correspond to an altered CD4^+^ T-cell activation status in TB (70).

No genes were SDE when contrasting TB *vs.* TBI in our datasets before or upon stimulation. Only when applying less stringent criteria (absolute logFC >0.58 and adjusted p-value <0.05) we observed a modest increase upon ESAT6-CFP10 stimulation in gene transcripts mostly involved in innate immunity (*MSR1/CD204*, *FCGR1B* and *C*) (**Supplementary Figure 9B**), while the expression of *GZMK*, a gene encoding granzyme Z, a serine protease mediating lymphocyte and NK cell cytotoxic action, was modestly reduced upon PPD stimulation (**Supplementary Figure 9C**). Studies conducted in stimulated PBMCs previously, reported a strong enrichment of B-cell immunity pathways in TB (24, 26) when comparing those to TBI individuals. Differences among the age groups (adult *vs.* pediatric population) in addition to different stimulant dosage and duration used may account for such discrepancies. Notably, although the studies in adult patients used high throughput transcriptomic techniques to examine the gene expression profiles of *Mtb*-specific antigen-stimulated PBMCs (25, 26, 30), the focus of these studies was more towards identifying TB biomarkers rather than providing insights into TB immunopathology.

The very limited number of SDE genes between TB *vs.* TBI or TB *vs.* HC could be due to the lack of a neutrophil population in the PBMC samples. Berry at al. (71). have previously reported that the differential gene expression profile in peripheral whole blood was neutrophil driven when comparing TB *vs.* TBI and HC in an adult cohort. Therefore, the performance of this test needs to be studied further and particularly in populations with high TB prevalence where a high proportion of children with pneumonia may already have been infected through previous TB exposure. This is a limitation also common to the IGRA tests. However, an important finding was the fact that *in vitro* stimulation with the use of ESAT-6 and CFP-10 considerably increased the number of differentially expressed genes in patients with TB vs those with pneumonia. Therefore, in future biomarker studies the diagnostic performance may considerably improve with the use of whole blood samples stimulated overnight with the ESAT6-CFP10 proteins *in vitro* before the sample is examined in an RT-PCR system. Although no genes crossed significance and LFC thresholds when comparing TB *vs.* TBI upon stimulation, there are some genes with smaller LFC that appear upon stimulation and could be studied further in larger populations and especially in settings where TB is endemic.

In our search for diagnostic markers, we used a training and a test dataset to identify molecular signatures able to discriminate TB disease from pneumonia. Our study findings add to the existing knowledge on genes able to discriminate TB from pneumonia in stimulated samples (*PID1* as a single gene upon ESAT6-CFP10 stimulation, as well as the combination of *STAT1* and *IFI44* upon PPD stimulation) with excellent performance. A 2-gene signature (*ADA* and *HIST2H2AA3*) can distinguish TB from pneumonia in unstimulated samples with an AUC of 76.8% (CI_95%_ 50.5%-100.0%) in the test set. Although the CIs are wide, and robust conclusions cannot be made, this could be highlighting the role of neutrophil derived genes in other signatures that achieve higher performance in unstimulated samples. The single gene signature (*PID1)* identified in the post ESAT6-CFP10 stimulation samples performed best and achieved an AUC of 100% (CI_95%_ 28.0%-100.0%) in comparison to an AUC of 91.7% (CI_95%_ 75.0%-100%) for PPD stimulated samples. PPD is a multiple protein mixture and could, therefore, be promoting a more diverse immune response. This fact, together with the pronounced ability of our approach to reveal highly enriched pathway profiles characterizing the TB patients, underlines the pertinence of the current approach, which although laborious and not cost-effective, provides further insights into TB immunology and new potential for the TB biomarker discovery pathway (72). The potential benefit of stimulation is further illustrated by the performance of the Sweeney 3-gene signature (12) in our dataset. Despite the fact that the Sweeney signature was discovered on unstimulated whole blood RNA samples, it performed best in the stimulated PBMCs with ESAT6-CFP10, while performing poorly in the unstimulated PBMC samples. This suggests that stimulation enhances measurable immune response gene expression and compensates for the rather limited representation of cellular heterogeneity in PBMCs compared to whole blood (73), thereby improving the performance of signatures, even those discovered in unstimulated samples. Additionally, the good performance of the 3-gene signatures indicates that the difference in gene expression between cases and controls may be less dependent on the presence of neutrophils or other granulocytes.

As for the biomarker genes*, IFI44* is a member of the type I interferon stimulated gene (ISG) family with known antiviral response and inflammatory function on innate immunity. Upregulation of genes in the ISG family is accompanied by proinflammatory cytokines and chemokine release, which enhance *Mtb* clearance in human macrophages (67). The transcription factor STAT1, which becomes phosphorylated and activates ISGF3 forming part of the type I interferon response, has been previously identified, not unexpectedly, as a TB biomarker (54, 74), while *ADA* encodes an enzyme (adenosine deaminase) that regulates purine metabolism in lymphocytes and may serve as diagnostic tool in extrapulmonary TB (75). On the other hand, although histone modifications have been involved in TB pathophysiology (76), *HIST2H2AA3*, encoding a member of the core histone H2A family, has not been reported in TB biomarker studies. In contrast, *PID1/NYGGF4*, our single gene discriminator of TB versus pneumonia in ESAT6-CFP10 stimulated samples, has been reported as differentially expressed when comparing TB versus HC, or even TBI, in a similar setting in adult patients (29, 30). However, in contrast to our study, following analysis in a validation group *PID1* failed to enter a discriminating signature in the adult setting, likely reflecting differences in TB-specific PBMC responses between adult and pediatric patients. Although the contribution of *PID1* in TB is currently unknown, it is worth noting that its expression is potentially linked with chronic lung disease (77) while the rs1419958 polymorphism has been associated with pulmonary response to oxidative stress (78), suggesting that its differential expression may mark childhood TB-specific stress induced pathways.

ESAT6-CFP10 stimulation of PBMCs had previously identified *RETN* and *KLK1* as two genes that could discriminate TB from TBI (but also from HC) in adults with an AUC of 84.4% and 83.3% in qPCR validation, respectively (30). Li and colleagues (79) reported that a single gene (IL-9 mRNA) can separate children with TB from healthy controls with an AUC of 92% after ESAT-6 stimulation, while Jenum and colleagues (31) reported that an 8-gene biomarker signature separated children with TB from asymptomatic siblings in stimulated blood with an AUC of 88%.

Our study has several limitations. Firstly, the study included a modest sample size, which reflects the low prevalence of TB in our recruitment setting. Given that different settings and populations, such as areas with varying TB prevalence rates and differences in genetic backgrounds, may introduce significant variability in gene expression patterns, further validation in a large-scale multi-site cohort is essential. Moreover, an RT-PCR-based validation step would provide additional confidence in the findings. Given the Mtb stimulation step, it is of particular importance to assess the performance of the signatures in patients with non-TB pneumonia who may have been exposed to TB in the past. It is noteworthy that our study focused on peripheral blood mononuclear cells (PBMCs) and successfully identified sparse diagnostic signatures for TB with excellent performance. However, we were unable to assess the role and contribution of neutrophils, which play a key role in host response to Mtb, as they are not present in PBMCs. Finally, while most studies on RNA TB biomarkers focus on RNA derived from whole blood, our findings suggest that the additional steps required for PBMC isolation and antigen stimulation are justified by the excellent performance of these RNA signatures. Although some of these processes are laborious and time-consuming, future technological advancements may simplify and streamline these steps making our signatures suitable for translation into a near point-of-care test.

## Data Availability

The datasets presented in this study can be found in online repositories. The names of the repository/repositories and accession number(s) can be found below: GSE249575 (GEO).
