# Supplementary material for: Novel RNA biomarkers improve discrimination of children with tuberculosis disease from those with non-TB pneumonia after in vitro stimulation
Source: Front Immunol. 2024 Sep 26;15:1401647. doi: 10.3389/fimmu.2024.1401647 (PMC11464340; doi:10.3389/fimmu.2024.1401647)
Supplement: Supplementary file 1 [file DataSheet1.docx]

Supplementary Material

# Supplementary Data

NA

# Supplementary Figures and Tables

## Supplementary Figures


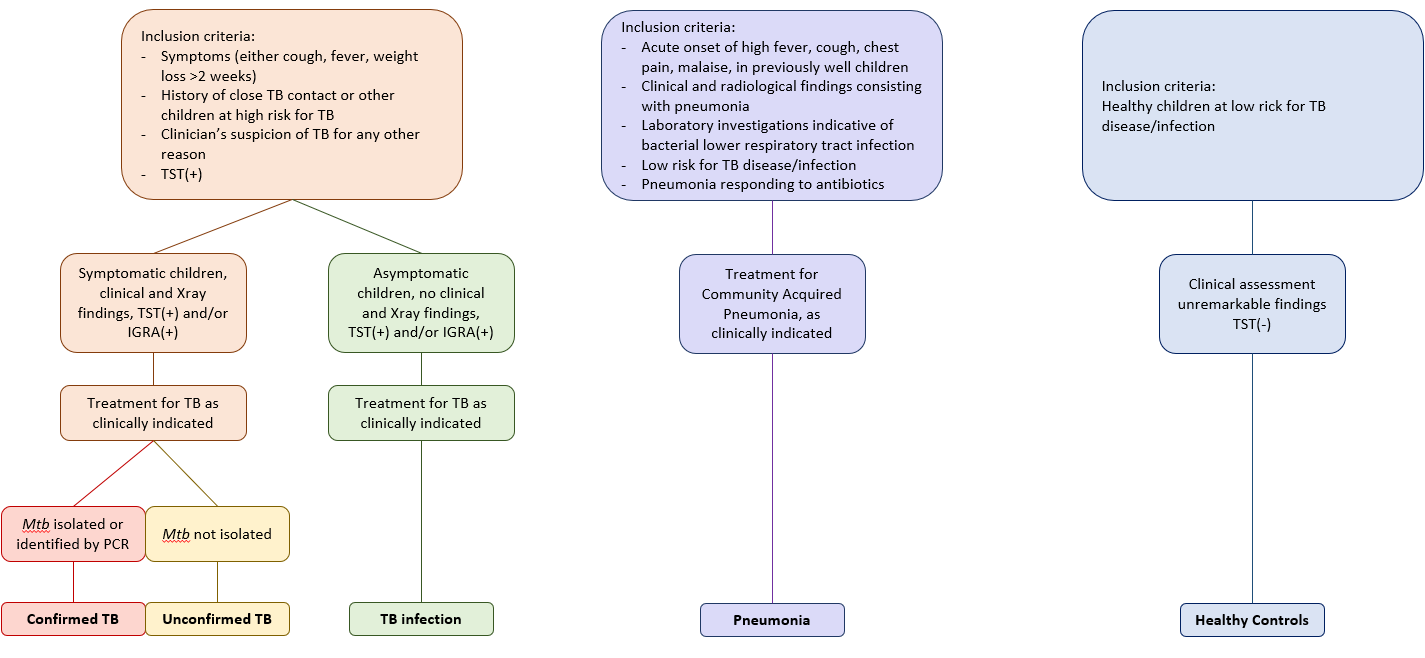


**Supplementary Figure 1.** Diagnostic algorithm.


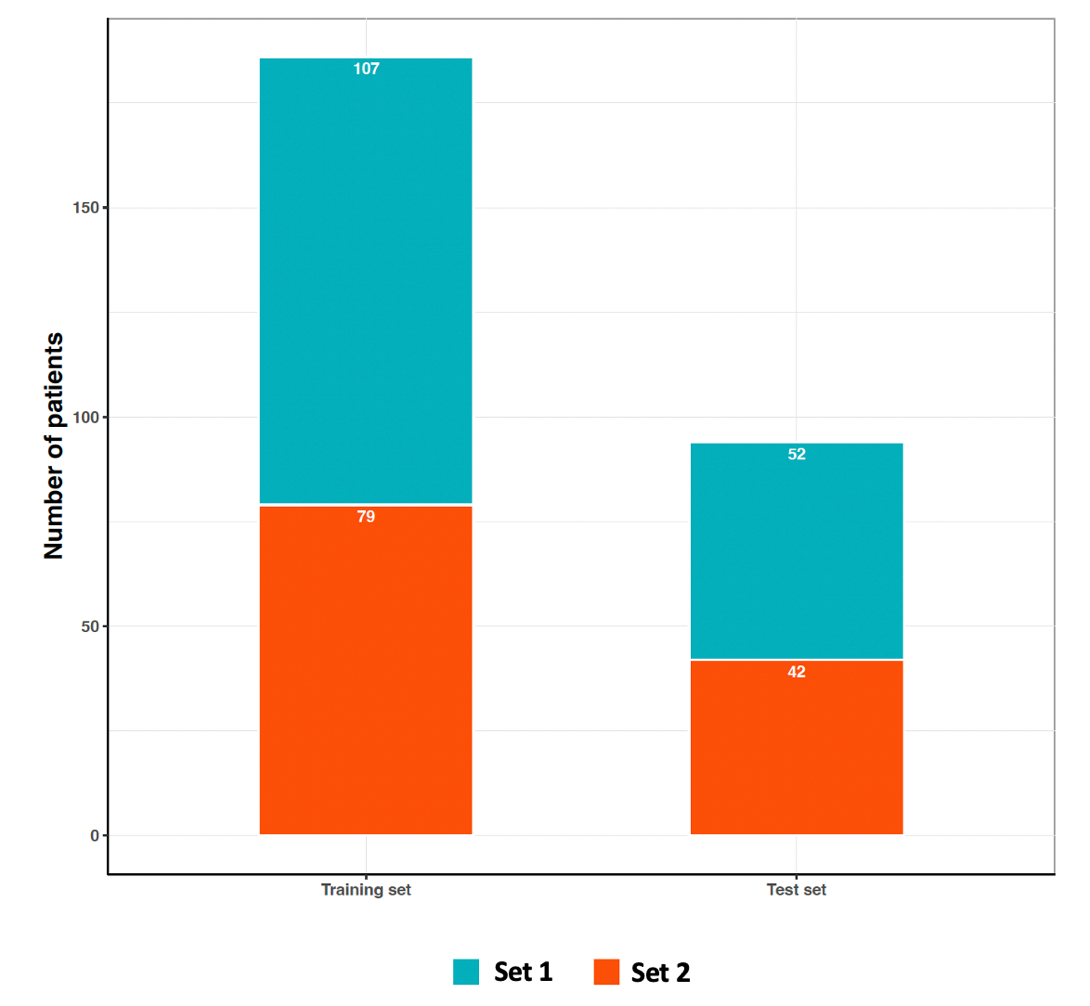


**Supplementary Figure 2.** Number of samples from set 1 and set 2 in the training and validation sets after random split (70% and 30%)


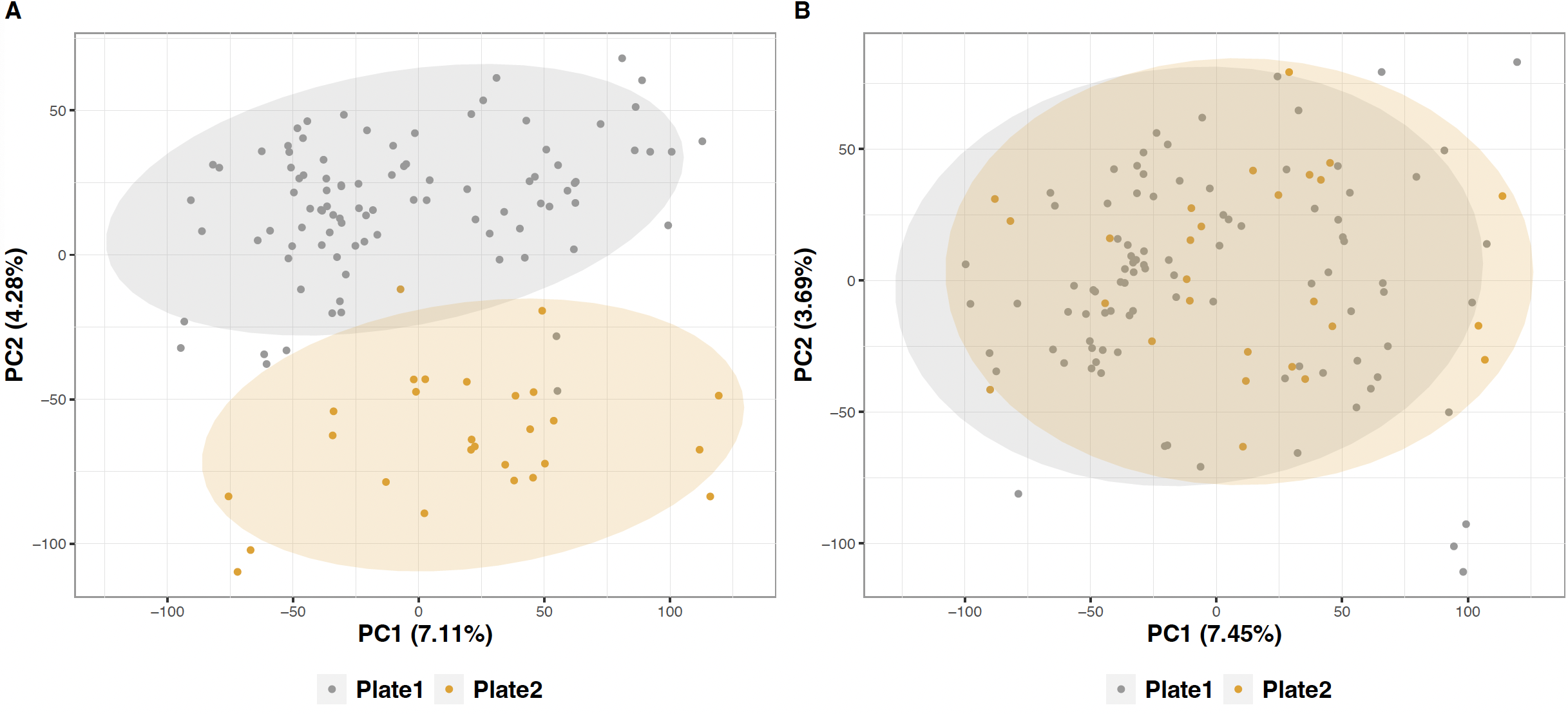


**Supplementary Figure 3.** PCA plot before (**A**) and after (**B**) plate-correction in the second set.

**
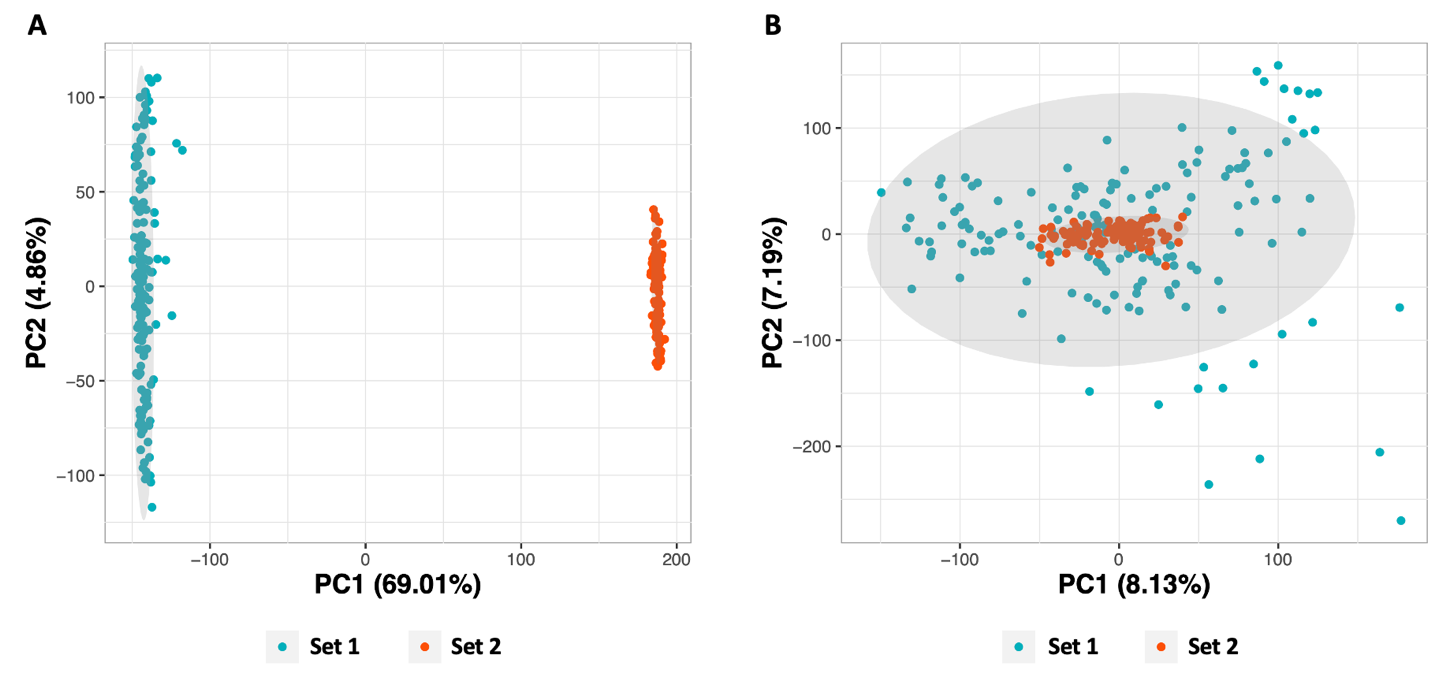
**

**Supplementary Figure 4.** PCA plot before (**A**) and after (**B**) batch-correction.


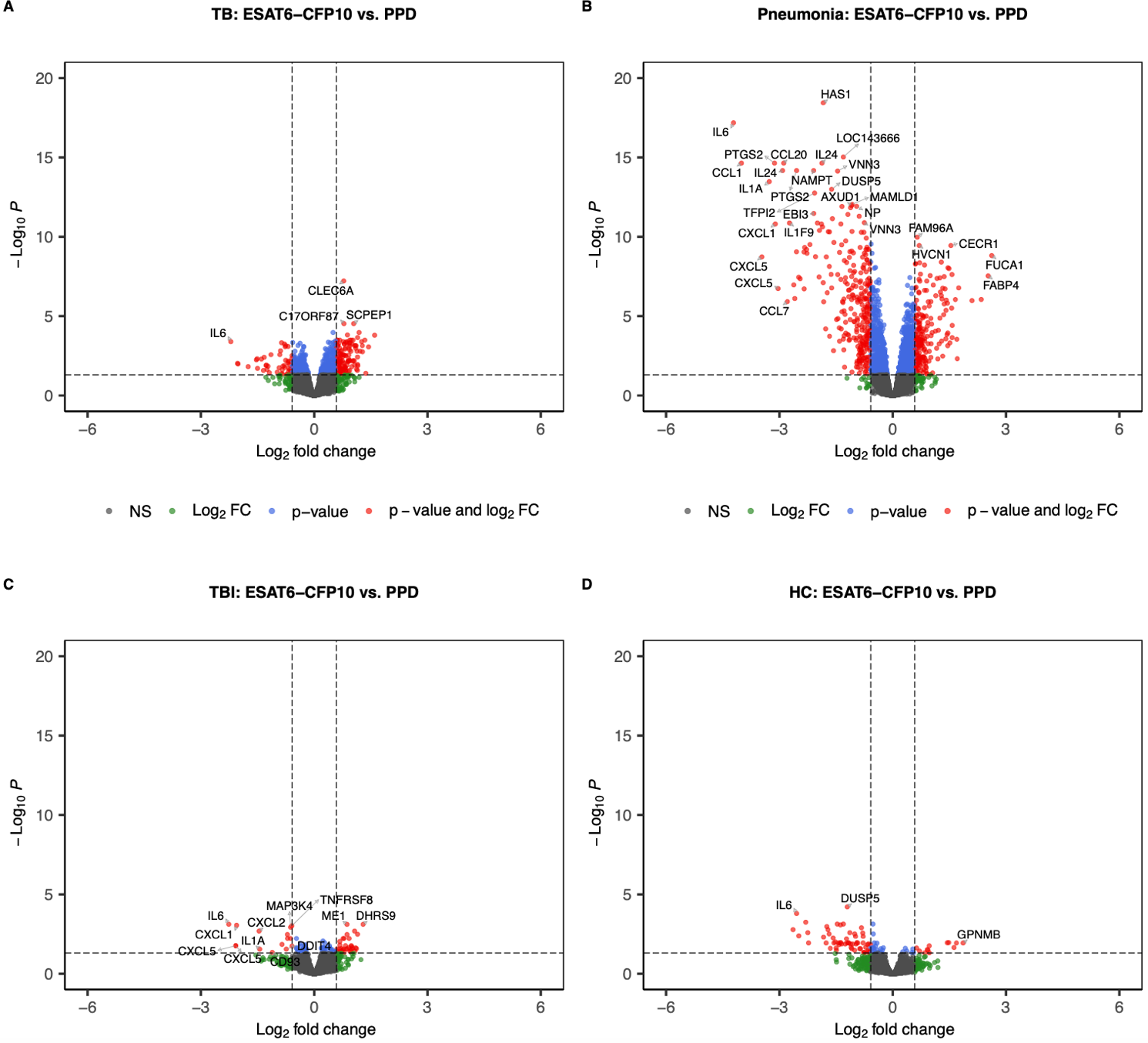


**Supplementary Figure 5.** Volcano plots showing log2 Fold-Change (LFC) and -log10 adjusted p-values from differential expression analysis comparing ESAT6-CFP10 *vs.* PPD stimulated samples in TB (**A**), Pneumonia (**B**), TBI (**C**) and HC (**D**). Red dots represent the genes with adjusted p-value < 0.05 and absolute LFC > 0.58; blue dots represent the genes with adjusted p-values < 0.05 and absolute LFC < 0.58; green dots represent the genes with adjusted p-values > 0.05 and absolute LFC > 0.58; black dots represent the non-significant genes (adjusted p-values > 0.05 and absolute LFC < 0.58).


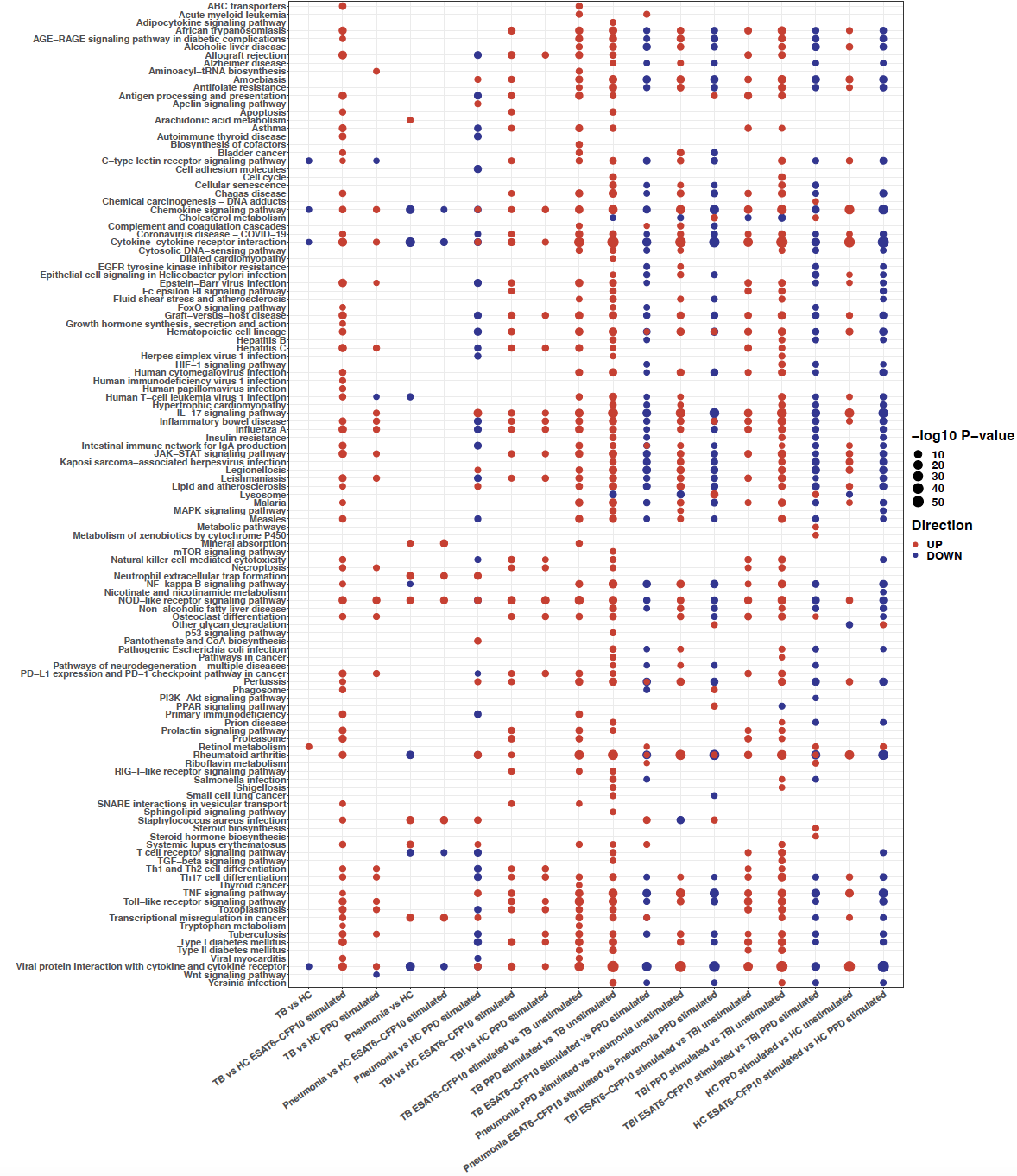


**Supplementary Figure 6**: Significant pathways with p-value < 0.05 (columns) up and downregulated for each disease group comparisons (rows) in the whole cohort.


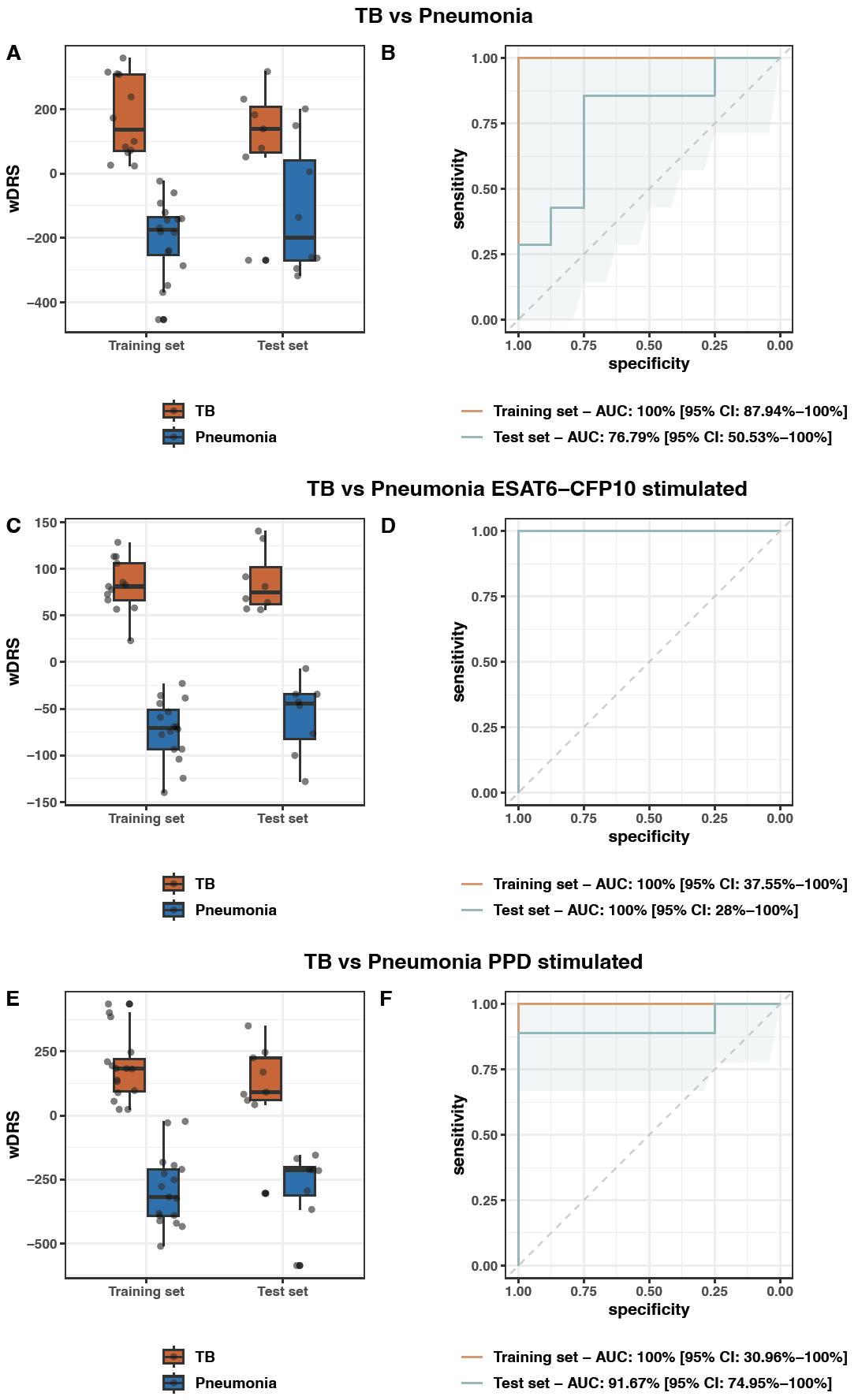


**Supplementary Figure 7.** Boxplots and ROC curves of the weighted disease risk score of the signatures to distinguish TB from Pneumonia in unstimulated samples (**A**, **B**), ESAT6-CFP10 stimulated samples (**C**, **D**) and PPD stimulated samples (**E**, **F**) in training and validation sets. A 2-gene signature (*ADA* and *HIST2H2AA3*) identified in the unstimulated samples achieved an AUC of 100.0% (CI_95%_ 87.9%-100.0%), sensitivity of 100.0% and specificity of 100.0% in the training set, and an AUC of 76.8% (CI_95%_ 50.5%-100.0%), sensitivity of 85.7% (CI_95%_ 57.1%-100.0%) and specificity of 75.0% (CI_95%_ 35.7%-100.0%) in the test set (**A**, **B**). 1 gene (*PID1*) achieved an AUC of 100% (CI_95%_ 37.6%-100.0%), sensitivity of 100.0% and specificity of 100.0% in the training set of the ESAT6-CFP10 stimulated samples, and an AUC of 100.0% (95% CI: 28.0%-100.0%), sensitivity of 100.0% and specificity of 100.0% in the test set (**C**, **D**). A 2-gene signature (*STAT1* and *IFI44*) identified in the PPD stimulated samples achieved an AUC of 100.0% (CI_95%_ 31.0%-100.0%), sensitivity of 100.0% and specificity of 100.0% in the training set, and an AUC of 91.7% (CI_95%_ 75.0%-100.0%), sensitivity of 88.9% (CI_95%_ 62.5%-100.0%) and specificity of 100.0% in the test set (**E**, **F**).


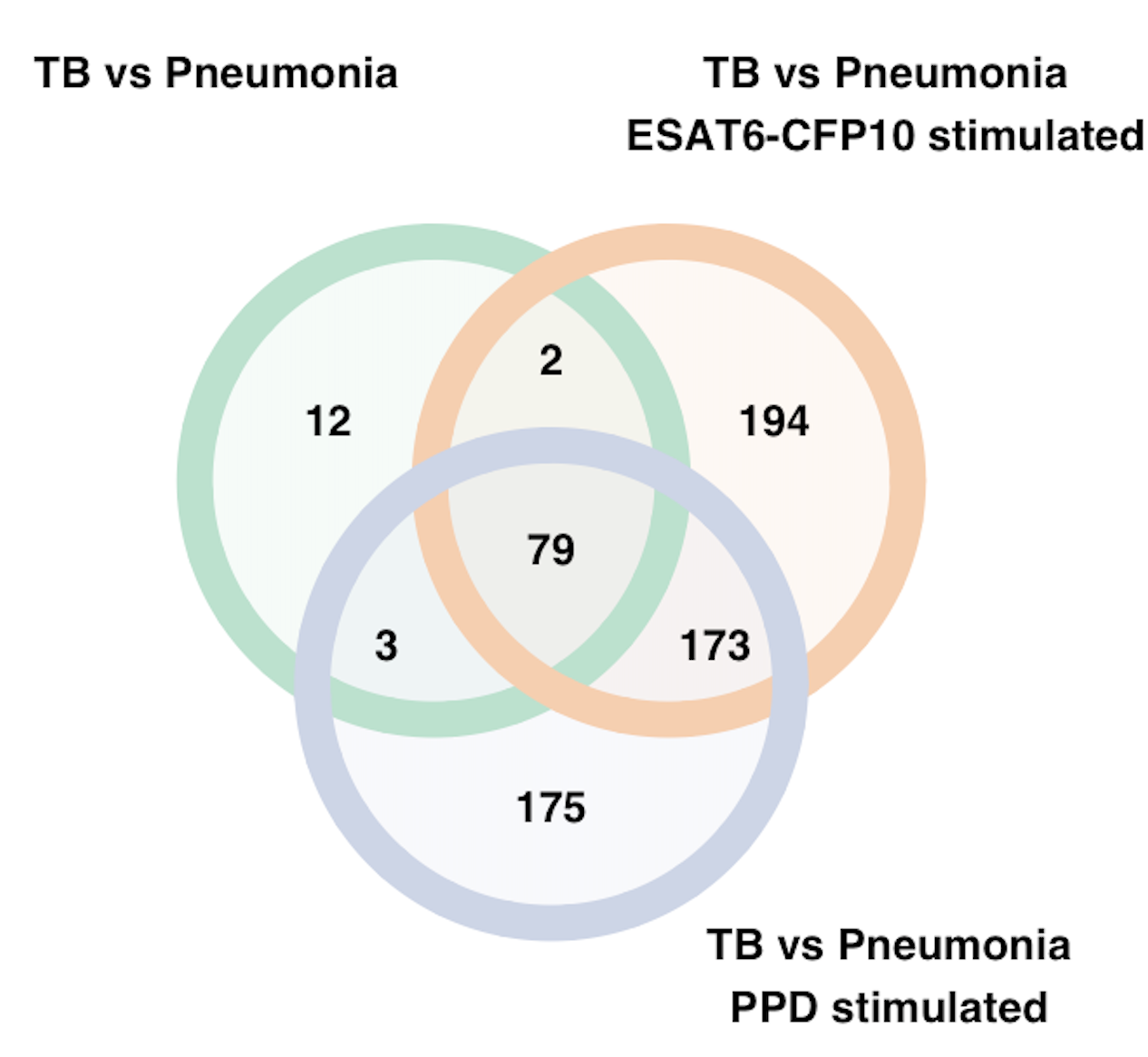


**Supplementary Figure 8.** Venn diagram showing the intersection of SDE genes when contrasting TB versus pneumonia for unstimulated (green circle), ESAT6-CFP10 stimulated (orange circle) and PPD (purple circle) stimulated samples.

##
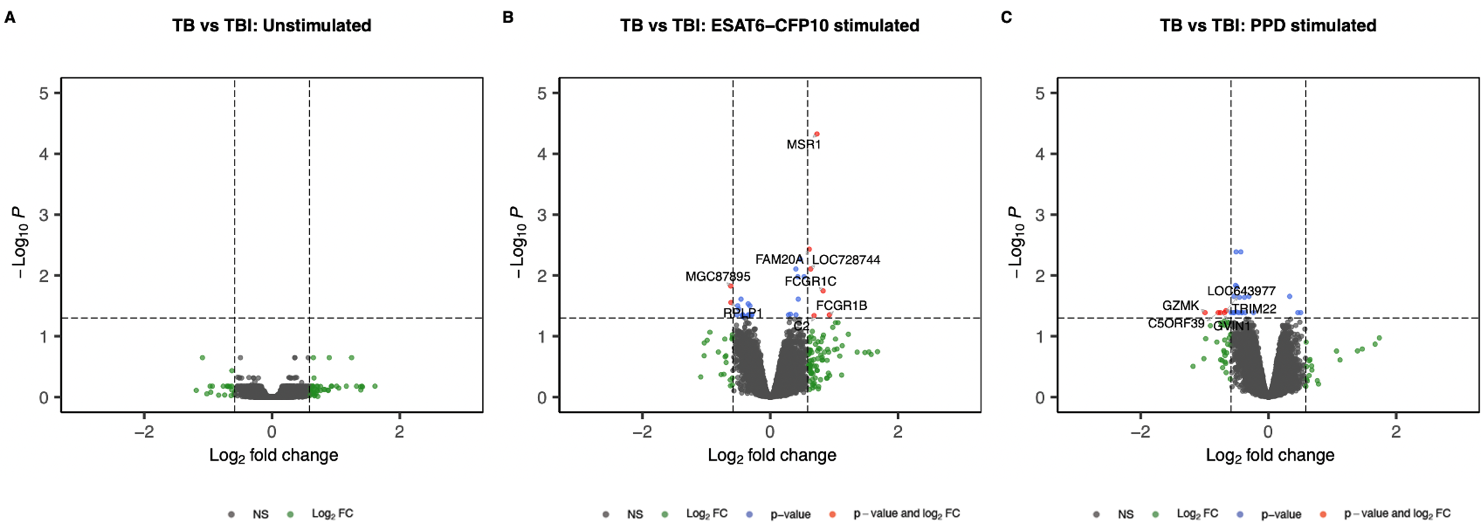


**Supplementary Figure 9.** Volcano plots showing log2 Fold-Change (LFC) and -log10 adjusted p-values from differential expression analysis comparing TB *vs.* TBI in the unstimulated samples (**A**), in the ESAT6-CFP10 stimulated samples (**B**) and in the PPD stimulated samples (**C**). Red dots represent the genes with adjusted p-value < 0.05 and absolute LFC > 0.58; blue dots represent the genes with adjusted p-values < 0.05 and absolute LFC < 0.58; green dots represent the genes with adjusted p-values > 0.05 and absolute LFC > 0.58; black dots represent the non-significant genes (adjusted p-values > 0.05 and absolute LFC < 0.58).

## Supplementary Tables

|  | **N (%)** | **N=25 (24.5%)** | **N=27 (26.5%)** | **N=25**  **(24.5%)** | **N=25**  **(24.5%)** |
| --- | --- | --- | --- | --- | --- |
| **Disease** | **Overall** | **TB** | **TBI** | **Pneumonia** | **Controls** |
| **Age (years) median (IQR)** | 5.6 (1.4-12.4) | 4.5 | 7.38 | 5.2 | 5.36 |
| **Females** | 51 (50%) | 10 | 13 | 15 | 13 |
| **Child’s Birth Place** |  |  |  |  |  |
| Greece | 85 (82%) | 23 | 20 | 20 | 22 |
| Albania | 7 (6.8%) | 0 | 0 | 4 | 3 |
| Russian Federation | 2 (2%) | 0 | 1 | 1 | 0 |
| Eastern Europe | 2 (2%) | 0 | 2 | 0 | 0 |
| Asia | 5 (5%) | 2 | 3 | 0 | 0 |
| Africa | 1 (1%) | 0 | 1 | 0 | 0 |
| **Minority (Roma)** | 4 (4%) | 1 | 2 | 1 | 0 |
| **Immigrant status** | 38 (37%) | 14 | 16 | 5 | 3 |
| **BCG vaccinated** | 8 (7.8%) | 4 | 4 | 0 | 0 |
| **TST** | 48 (47%) | 21* | 27 | 0 | 0 |
| **<10mm** |  | 1 | 1 |  |  |
| **10-15mm** |  | 9 | 9 |  |  |
| **>15** |  | 9 | 17 |  |  |

*4 patients were TST(-)

**Supplementary Table 1.** Demographic and clinical characteristics of subjects enrolled (N=102)

| Type of  TB disease | Intrathoracic  N=19 | Pulmonary infiltrate | 12 |
| --- | --- | --- | --- |
|  |  | Hilar adenopathy | 5 |
|  |  | Pulmonary infiltrate and hilar adenopathy | 17 |
|  |  | Pleural effusion and pulmonary infiltrate | 2 |
|  | Mixed  Intra- and extra-thoracic  N=4 | Meningitis | 3 |
|  |  | Peripheral Lymphadenopathy | 1 |
|  | Extrathoracic  N=2 | Osteomyelitis | 1 |
|  |  | Meningitis | 1 |
| Symptoms | Yes | Fever | 14 |
|  |  | Cough | 10 |
|  |  | Anorexia | 5 |
|  |  | Vomiting | 4 |
|  |  | Skeletal pain | 1 |
| Microbiological  TB  confirmation | No |  | 0 |
|  | Yes | Culture pos | 12 |
|  |  | XPert Mtb/RIF pos | 4 |
|  |  | Xpert MTB/RIF and culture pos | 9 |

**Supplementary Table 2.** Clinical and laboratory characteristics of the 25 patients with TB disease

|  | **Disease** | **Stimulant** | **Number** | **Age (years) median IQR** | **Sex (male, %)** |
| --- | --- | --- | --- | --- | --- |
| **TRAINING SET** | Confirmed TB | NON | 12 | 3.2 (2.5-10.1) | 50 |
|  |  | CFP10-ESAT6 | 13 | 2.0 (1.1-10.8) | 69 |
|  |  | PPD | 16 | 2.0 (1.1-5.3) | 56 |
|  | Pneumonia | NON | 16 | 4.2 (1.8-6.1) | 50 |
|  |  | CFP10-ESAT6 | 16 | 6.0 (5.1-8.2) | 38 |
|  |  | PPD | 17 | 4.8 (1.6-7.0) | 29 |
|  | TBI | NON | 16 | 8.1 (5.0-8.9) | 50 |
|  |  | CFP10-ESAT6 | 17 | 8.2 (6.0-10.0) | 47 |
|  |  | PPD | 18 | 7.5 (5.1-8.8) | 50 |
|  | Healthy Controls | NON | 15 | 4.0 (3.0-5.8) | 47 |
|  |  | CFP10-ESAT6 | 13 | 5.5 (3.0-7.0) | 54 |
|  |  | PPD | 17 | 5.5 (4.0-6.0) | 47 |
| **TEST**  **SET** | Confirmed TB | NON | 7 | 2.0 (1.9-7.9) | 100 |
|  |  | CFP10-ESAT6 | 8 | 3.1 (1.8-6.1) | 62 |
|  |  | PPD | 9 | 3.0 (1.8-5.0) | 67 |
|  | Pneumonia | NON | 8 | 5.8 (4.0-6.5) | 25 |
|  |  | CFP10-ESAT6 | 8 | 1.7 (1.5-3.8) | 50 |
|  |  | PPD | 8 | 5.6 (3.9-5.8) | 62 |
|  | TBI | NON | 8 | 7.5 (6.0-9.2) | 62 |
|  |  | CFP10-ESAT6 | 8 | 7.0 (5.0-8.2) | 62 |
|  |  | PPD | 8 | 8.3 (6.5-10.2) | 62 |
|  | Healthy Controls | NON | 8 | 5.8 (5.4-7.0) | 50 |
|  |  | CFP10-ESAT6 | 6 | 5.2 (4.2-5.9) | 17 |
|  |  | PPD | 8 | 5.2 (3.8-6.0) | 50 |

**Supplementary Table 3.** Number, median age in years, interquartile range (IQR), and percentage of males in each disease group for the training and test sets.

|  | Training set | | | Test set | | |
| --- | --- | --- | --- | --- | --- | --- |
|  | *ADA* and *HIST2H2AA3* | | | *ADA* and *HIST2H2AA3* | | |
|  | AUC | Sensitivity | Specificity | AUC | Sensitivity | Specificity |
| Unstimulated samples | 100% (87.9%-100%) | 100% | 100% | 76.8% (50.5%-100%) | 85.7% (57.1%-100%) | 75.0% (35.7%-100%) |
| ESAT6-CFP10 stimulated samples | 96.15%(88.37%-100%) | 92.31% (76.92%-100%) | 100% | 98.44% (94.11%-100%) | 100% | 87.5% (62.5%-100%) |
| PPD stimulated samples | 81.99% (66.02%-97.95%) | 81.25% (62.5%-100%) | 88.24% (70.59%-100%) | 91.67% (78.24%-100%) | 88.89% (66.67%-100%) | 87.5% (62.5%-100%) |
|  | *PID1* | | | *PID1* | | |
|  | AUC | Sensitivity | Specificity | AUC | Sensitivity | Specificity |
| Unstimulated samples | 79.17% (61.42%-96.92%) | 75% (50%-100%) | 75% (50%-93.75%) | 71.43% (39.51%-100%) | 57.14% (28.57%-85.71%) | 100% |
| ESAT6-CFP10 stimulated samples | 100% (37.6%-100%) | 100% | 100% | 100% (28.0%-100%) | 100% | 100% |
| PPD stimulated samples | 89.34% (76.59%-100%) | 93.75% (81.25%-100%) | 82.35% (64.71%-100%) | 98.61% (94.76%-100%) | 88.89% (66.67%-100%) | 100% |
|  | *STAT1* and *IFI44* | | | *STAT1* and *IFI44* | | |
|  | AUC | Sensitivity | Specificity | AUC | Sensitivity | Specificity |
| Unstimulated samples | 75.52% (52.62%-98.42%) | 75% (50%-100%) | 87.5% (68.75%-100%) | 69.64% (39.34%-99.94%) | 71.43% (28.57%-100%) | 75% (37.5%-100%) |
| ESAT6-CFP10 stimulated samples | 91.83% (79.29%-100%) | 92.31% (76.92%-100%) | 87.5% (68.75%-100%) | 93.75% (80.65%-100%) | 100% | 87.5% (62.5%-100%) |
| PPD stimulated samples | 100% (31.0%-100%) | 100% | 100% | 91.7% (75.0%-100%) | 88.9% (62.5%-100%) | 100% |

**Supplementary Table 4.** Performance of the TB vs Pneumonia signatures discovered in each of the stimulus-specific subgroups on all subgroups. The stimulus-specific subgroup that the signature was discovered on is highlighted in green. The numbers in brackets denote 95% confidence intervals.

|  | Training set | | | Test set | | |
| --- | --- | --- | --- | --- | --- | --- |
|  | GBP5, DUSP3 and KLF2 | | | GBP5, DUSP3 and KLF2 | | |
|  | AUC | Sensitivity | Specificity | AUC | Sensitivity | Specificity |
| Unstimulated samples | 49.48% (25.22%-73.73%) | 41.67% (16.67%-66.67%) | 81.25% (62.5%-100%) | 64.29% (32.1%-96.47%) | 42.86% (0%-85.71%) | 100% |
| ESAT6-CFP10 stimulated samples | 89.9% (78.3%-100%) | 92.31% (76.92%-100%) | 75% (56.25%-93.75%) | 89.06% (67.41%-100%) | 87.5% (62.5%-100%) | 100% |
| PPD stimulated samples | 82.35% (67.7%-97%) | 100% (100%-100%) | 52.94% (29.41%-76.47%) | 86.11% (66.01%-100%) | 66.67% (33.33%-100%) | 100% |

**Supplementary Table 5.** Performance of the Sweeney TB signature on our Training and Test sets of unstimulated and stimulated samples.

| **Gene Name** | **Probe ID** | **Gene Name** | **Probe ID** | **Gene Name** | **Probe ID** |
| --- | --- | --- | --- | --- | --- |
| ADA | 7210192 | GNLY | 5360064 | MT1G | 1170300 |
| ALAS2 | 4180768 | GNLY | 6580041 | MT1H | 130093 |
| ANPEP | 5810612 | GZMA | 3420612 | MT1X | 6620528 |
| AQP9 | 160494 | GZMB | 1850523 | OLFM4 | 6770131 |
| ARG1 | 5270753 | GZMH | 2370010 | OLR1 | 7510132 |
| BPI | 4200746 | H1F0 | 630278 | ORM1 | 2850315 |
| C19ORF59 | 1070367 | HIST1H1C | 5570279 | PADI4 | 2650035 |
| CAMP | 5860075 | HIST1H4H | 2970019 | PGLYRP1 | 6250615 |
| CD14 | 7000369 | HLA-DPA1 | 1190039 | PI3 | 1050168 |
| CD14 | 6370369 | HLA-DQA1 | 780403 | PID1 | 1410114 |
| CEACAM6 | 7400377 | HOPX | 3850246 | PRF1 | 4670193 |
| CEACAM8 | 4780075 | HS.407903 | 5720768 | RETN | 4610129 |
| CTSG | 1500735 | HTRA1 | 730612 | RNASE2 | 2640068 |
| CX3CR1 | 2070168 | INDO | 380259 | RNASE3 | 3130543 |
| CXCL10 | 6270553 | KLRB1 | 6200019 | RPL14 | 3930008 |
| CXCL9 | 5570278 | KLRD1 | 1770598 | S100A12 | 1410221 |
| DEFA1 | 4540239 | LCN2 | 4390398 | S100A4 | 5290270 |
| DEFA1B | 7150170 | LOC100129685 | 7560215 | S100A4 | 3990458 |
| DEFA1B | 4860128 | LOC100133583 | 3450736 | S100A8 | 6060468 |
| DEFA1B | 870477 | LOC100133678 | 5310224 | S100A9 | 5390220 |
| DEFA3 | 2970747 | LOC653156 | 4010180 | S100P | 1510424 |
| DEFA4 | 6550164 | LRRN3 | 2370041 | SEPTIN5 | 6960022 |
| DNER | 4570669 | LRRN3 | 2230538 | SERPINA1 | 730047 |
| ELANE | 7650497 | MAP1LC3A | 5490240 | SIGLEC10 | 4480497 |
| FCER1A | 3360615 | METTL7B | 5310142 | SLPI | 2140707 |
| FPR1 | 1030270 | MPO | 3520601 | SOD2 | 3890326 |
| GBP1 | 2190148 | MS4A3 | 5050544 | STAT1 | 4810187 |
| GBP1 | 6840035 | MS4A7 | 3840564 | STAT1 | 2570079 |
| GBP2 | 1940162 | MS4A7 | 990328 | STAT1 | 1820750 |
| GBP4 | 1980524 | MT1A | 6200402 | TCL1A | 70110 |
| GBP5 | 1510364 | MT1E | 2070288 | TGFBR3 | 3190379 |
| GNG11 | 1580025 | MT1F | 4220672 | VCAN | 5910113 |

**Supplementary Table 6.** List of SDE genes when contrasting TB versus pneumonia for unstimulated.

| **Gene Name** | **Probe ID** | **Gene Name** | **Probe ID** | **Gene Name** | **Probe ID** |
| --- | --- | --- | --- | --- | --- |
| AADACL1 | 7210327 | GCH1 | 1010360 | MS4A6A | 1770152 |
| AADACL1 | 7650020 | GJB2 | 5260095 | MS4A7 | 990328 |
| ACOT7 | 4290050 | GLIPR1 | 6220746 | MS4A7 | 3840564 |
| ADA | 7210192 | GLT25D1 | 7380279 | MT1F | 4220672 |
| ADAP2 | 3990433 | GNG11 | 1580025 | MT1G | 1170300 |
| ADORA3 | 870138 | GNLY | 5360064 | MT1H | 130093 |
| ADORA3 | 6580482 | GNLY | 6580041 | MUC1 | 7650026 |
| AGPAT9 | 2060477 | GPNMB | 1570154 | MX1 | 1690066 |
| AIF1 | 3800047 | GPNMB | 6840164 | MXD1 | 670086 |
| AIF1 | 1940047 | GPR109A | 3830228 | NCR3 | 3800019 |
| AIM2 | 1260270 | GPR109B | 3180220 | NFXL1 | 1230731 |
| ALAS2 | 4180768 | GPR162 | 3520598 | NISCH | 5960678 |
| ALDH2 | 840253 | GPX1 | 60647 | NKG7 | 2450427 |
| ALOX5 | 1780273 | GRN | 6840184 | NLRP12 | 520470 |
| ANG | 3930392 | GRN | 1940021 | NPL | 540243 |
| ANKRD22 | 4150270 | GZMA | 3420612 | NT5DC2 | 6840301 |
| ANKRD22 | 130181 | GZMB | 1850523 | NUP214 | 4850164 |
| ANKRD33 | 4860707 | GZMH | 2370010 | OAS2 | 7320561 |
| ANPEP | 5810612 | GZMK | 1260482 | OAS2 | 2230204 |
| AP1S2 | 1070192 | H1F0 | 630278 | OAS3 | 990768 |
| AP1S2 | 940706 | H2AFY | 3930154 | OLFM4 | 6770131 |
| APOBEC3G | 1190026 | HAPLN3 | 4900086 | OLFML2B | 7150019 |
| APOBEC3G | 4120369 | HCK | 6270022 | OLIG1 | 2030403 |
| APOC1 | 3830465 | HDC | 6280326 | OLIG2 | 1780239 |
| APOL3 | 1980750 | HEXB | 3800139 | OLR1 | 7510132 |
| AQP9 | 160494 | HIST1H1C | 5570279 | ORM1 | 2850315 |
| ASGR1 | 2370064 | HLA-DMA | 540563 | P2RX7 | 3370086 |
| ATF3 | 4780128 | HLA-DOA | 4200452 | PADI4 | 2650035 |
| ATG7 | 5220754 | HLA-DPA1 | 1190039 | PARP12 | 2340072 |
| ATP1B1 | 4220468 | HLA-DPB1 | 1050360 | PARP14 | 4150692 |
| ATP1B1 | 4180452 | HLA-DQA1 | 780403 | PARP9 | 5700735 |
| AVPI1 | 990500 | HLA-DQB1 | 7160474 | PARP9 | 3140707 |
| BASP1 | 2140121 | HLA-DRA | 2570564 | PCOLCE2 | 4810445 |
| BATF2 | 4730059 | HLA-DRA | 270168 | PDGFC | 4850327 |
| BATF3 | 5900253 | HLA-DRB3 | 1770504 | PDLIM7 | 4560164 |
| BHLHB2 | 2640735 | HLA-DRB4 | 510079 | PECAM1 | 430747 |
| BIRC3 | 5080021 | HLA-DRB4 | 7330398 | PGLYRP1 | 6250615 |
| BPI | 4200746 | HLA-DRB6 | 4200725 | PI3 | 1050168 |
| C10ORF54 | 6620292 | HLA-DRB6 | 620544 | PID1 | 1410114 |
| **Gene Name** | **Probe ID** | **Gene Name** | **Probe ID** | **Gene Name** | **Probe ID** |
| C17ORF60 | 4290056 | HNMT | 3870102 | PLIN2 | 460204 |
| C19ORF59 | 1070367 | HOPX | 3850246 | PLIN2 | 1400446 |
| C1ORF162 | 7050719 | HPSE | 630619 | PMAIP1 | 2750367 |
| C1QB | 5910019 | HS.133181 | 2710020 | PNKD | 4730349 |
| C20ORF123 | 5290196 | HS.407903 | 5720768 | PPARG | 830019 |
| C3AR1 | 840068 | HS.543887 | 6840477 | PRF1 | 4670193 |
| C4ORF18 | 3460014 | HS.72010 | 6760286 | PRIC285 | 5960343 |
| C4ORF18 | 2650605 | HS.99472 | 2470730 | PSMB8 | 7210326 |
| CAMP | 5860075 | HS3ST1 | 4560632 | PSMB8 | 3420632 |
| CARD9 | 3710685 | HSD11B1 | 5810132 | PSMB9 | 1940180 |
| CASP4 | 3850021 | HTRA1 | 730612 | PSME2 | 830403 |
| CCL1 | 1030204 | ICOS | 2070037 | PTGFRN | 2680411 |
| CCL3 | 6590682 | IDO1 | 5570711 | PTGFRN | 5550332 |
| CCL3L1 | 4250053 | IDO2 | 2600528 | PTGR1 | 6450440 |
| CCL3L1 | 610524 | IDO2 | 1190703 | PTGS1 | 1070450 |
| CCL3L3 | 2810010 | IER5L | 2710142 | PYGL | 2760427 |
| CCL4L2 | 2970575 | IFI27 | 3990170 | PYHIN1 | 7330538 |
| CCL8 | 6620121 | IFI35 | 2510220 | RAB32 | 2190128 |
| CCND2 | 3460520 | IFI44 | 2570300 | RARRES3 | 2650564 |
| CCND2 | 1570348 | IFI44L | 3870338 | RASSF2 | 5390095 |
| CD14 | 7000369 | IFIT2 | 2600747 | RASSF2 | 1980059 |
| CD14 | 6370369 | IFIT3 | 1500280 | RCN1 | 1500168 |
| CD163 | 2680092 | IFIT3 | 6510170 | RETN | 4610129 |
| CD163 | 20593 | IFIT3 | 520408 | RGS1 | 4490176 |
| CD163 | 7560487 | IFITM1 | 5360156 | RIN2 | 6590445 |
| CD1B | 6270202 | IFITM3 | 6650242 | RIPK2 | 4150520 |
| CD209 | 2640037 | IFNG | 630725 | RNASE1 | 4010296 |
| CD274 | 4900239 | IFNGR2 | 2570291 | RNASE1 | 1090307 |
| CD302 | 4640630 | IL15 | 870408 | RNASE2 | 2640068 |
| CD36 | 3310538 | IL15RA | 940356 | RNASE3 | 3130543 |
| CD36 | 1010592 | IL18RAP | 6520180 | RNASE6 | 130681 |
| CD40 | 6420520 | IL27 | 6520523 | RNF130 | 1510722 |
| CD69 | 2710575 | IL2RA | 130021 | RNU4-2 | 3850189 |
| CD69 | 6900634 | IL2RB | 290402 | RRAS | 4570670 |
| CD74 | 7040008 | IL3 | 6040332 | RSAD2 | 3360343 |
| CD74 | 1240070 | IL32 | 1400274 | RTN3 | 4920129 |
| CD74 | 4560047 | IL32 | 3440754 | RTP4 | 1450427 |
| CD9 | 6250487 | IMPA2 | 2340241 | S100A12 | 1410221 |
| CDKN1A | 6660382 | IMPDH1 | 3120431 | S100A4 | 3990458 |
| **Gene Name** | **Probe ID** | **Gene Name** | **Probe ID** | **Gene Name** | **Probe ID** |
| CEACAM6 | 7400377 | INDO | 380259 | S100A4 | 5290270 |
| CEACAM8 | 4780075 | IRF1 | 6250064 | S100A8 | 6060468 |
| CFB | 3370349 | IRF7 | 6400176 | S100A9 | 5390220 |
| CHST7 | 5900452 | IRG1 | 1770072 | S100P | 1510424 |
| CPVL | 60136 | IRS2 | 6110736 | SAMD9L | 3170136 |
| CPVL | 3870324 | ISG15 | 2100196 | SEPTIN5 | 6960022 |
| CSF1R | 1070014 | ISG20 | 6330132 | SERPING1 | 2030309 |
| CSF2 | 2750196 | ITGAV | 2690209 | SGK | 4390450 |
| CSF3R | 6270114 | ITGB5 | 2490411 | SGK1 | 1410209 |
| CTLA4 | 6400333 | ITGB5 | 2650114 | SGK1 | 2030324 |
| CTSG | 1500735 | KCTD12 | 5560500 | SIGLEC10 | 4480497 |
| CTSK | 1430278 | KIAA1598 | 5340162 | SIGLEC14 | 1400593 |
| CTSW | 6580408 | KIAA1618 | 7400743 | SLAMF1 | 5810136 |
| CXCL10 | 6270553 | KIR2DL3 | 3140242 | SLC16A10 | 7550192 |
| CXCL9 | 5570278 | KLRB1 | 6200019 | SLC27A1 | 6180608 |
| CYBASC3 | 6940152 | KLRD1 | 1770598 | SLC36A1 | 2940095 |
| CYBRD1 | 2370300 | KLRD1 | 3390594 | SLCO2B1 | 6580441 |
| CYBRD1 | 2450465 | LAG3 | 2680189 | SLCO2B1 | 1400121 |
| CYFIP1 | 4200343 | LAMP3 | 7320546 | SLPI | 2140707 |
| CYP27A1 | 7040735 | LAP3 | 3290292 | SMPDL3A | 4230669 |
| DBNDD2 | 6420168 | LCN2 | 4390398 | SOCS1 | 1990300 |
| DCUN1D3 | 7560288 | LGALS1 | 1450193 | SOCS2 | 6770673 |
| DDX60 | 7610053 | LGMN | 4560129 | STAB1 | 6040053 |
| DEFA1 | 4540239 | LGMN | 1300452 | STAT1 | 4810187 |
| DEFA1B | 7150170 | LILRA3 | 6110037 | STAT1 | 2570079 |
| DEFA1B | 4860128 | LILRA3 | 6200370 | STAT1 | 1820750 |
| DEFA1B | 870477 | LINCR | 580678 | STAT4 | 7320370 |
| DEFA3 | 2970747 | LMNB1 | 3420593 | SUCNR1 | 6560079 |
| DEFA4 | 6550164 | LOC100133583 | 3450736 | TAP1 | 7330392 |
| DLL1 | 4050687 | LOC100133678 | 5310224 | TAP2 | 3940477 |
| DNASE2 | 6200315 | LOC100133875 | 6330612 | TBXAS1 | 3940390 |
| DNER | 4570669 | LOC389386 | 4570164 | TCL1A | 70110 |
| DPYSL3 | 4290431 | LOC389386 | 4780044 | TFPI2 | 5080543 |
| DUSP5 | 5390161 | LOC400759 | 620403 | TGFBI | 7650358 |
| EBI2 | 4490520 | LOC441481 | 3840474 | TGM2 | 1580435 |
| EEPD1 | 50286 | LOC642567 | 5090382 | THBS1 | 5810685 |
| ELANE | 7650497 | LOC645638 | 70121 | TIMP1 | 7650433 |
| ENG | 4610753 | LOC648470 | 1450136 | TIMP2 | 780270 |
| EOMES | 7320372 | LOC649923 | 580445 | TKT | 6860202 |
| **Gene Name** | **Probe ID** | **Gene Name** | **Probe ID** | **Gene Name** | **Probe ID** |
| EPB41L3 | 1510538 | LOC652775 | 7610348 | TLR5 | 830440 |
| EPSTI1 | 5700725 | LOC728835 | 6560201 | TMEM119 | 3830762 |
| ERAP2 | 2260731 | LOC730249 | 4670114 | TMEM140 | 4670414 |
| ETV5 | 770746 | LOC730415 | 7210253 | TMEM158 | 3130220 |
| ETV7 | 6370768 | LOC730455 | 6510075 | TMEM170B | 4780491 |
| FABP4 | 150373 | LOC731682 | 10333 | TMEM51 | 380102 |
| FAM26F | 6590646 | LRP1 | 2710286 | TNF | 2640301 |
| FASLG | 4570612 | LRRC25 | 5310397 | TNFRSF4 | 650328 |
| FBXO6 | 3800398 | LTA | 1030743 | TNFSF10 | 870202 |
| FCER2 | 830152 | LTA4H | 1430524 | TNFSF13B | 4900435 |
| FCGR1A | 520086 | LTB | 5420091 | TNFSF13B | 6840020 |
| FCGR1B | 2710709 | LXN | 60670 | TPST1 | 4760747 |
| FCGR1C | 5570398 | M160 | 240220 | TRAFD1 | 1570129 |
| FCGRT | 4200176 | MAFB | 2060440 | TRIM22 | 5960747 |
| FCN1 | 6660398 | MAP1LC3A | 5490240 | TXN | 4290543 |
| FGL2 | 1430170 | MARCKS | 6060484 | UBD | 6420605 |
| FLJ22662 | 2140288 | MARCKSL1 | 6770242 | UBE2L6 | 2070170 |
| FOLR3 | 4010181 | MATK | 5670605 | UBE2L6 | 7000368 |
| FOS | 4280017 | MCOLN2 | 5490068 | VAMP5 | 2630195 |
| FPR1 | 1030270 | MERTK | 7550066 | VCAN | 5910113 |
| FSCN1 | 4560328 | MERTK | 4730315 | VMO1 | 1660484 |
| FTHL11 | 460164 | METTL7B | 5310142 | VNN1 | 5310754 |
| FTHL8 | 1980594 | MFSD1 | 510288 | VNN2 | 2690239 |
| FUCA1 | 2060121 | MGST3 | 7160400 | VNN2 | 7100161 |
| GAPT | 7570541 | MIR155HG | 5860722 | VSIG4 | 1410730 |
| GAPT | 6480079 | MNDA | 6290270 | VWF | 5870138 |
| GAS7 | 1070435 | MPO | 3520601 | WARS | 3710068 |
| GBP1 | 2190148 | MPP1 | 5310554 | WARS | 4860224 |
| GBP1 | 6840035 | MS4A14 | 840689 | XAF1 | 450189 |
| GBP2 | 1940162 | MS4A14 | 5810170 | ZMIZ1 | 2450131 |
| GBP4 | 1980524 | MS4A3 | 5050544 | ZNF467 | 2490333 |
| GBP5 | 1510364 | MS4A4A | 5890193 |  |  |
| GCH1 | 240053 | MS4A6A | 2260129 |  |  |

**Supplementary Table 7.** List of SDE genes when contrasting TB versus pneumonia for ESAT6-CFP10 stimulated.

| **Gene Name** | **Probe ID** | **Gene Name** | **Probe ID** | **Gene Name** | **Probe ID** |
| --- | --- | --- | --- | --- | --- |
| ABCA1 | 4060358 | GLIPR1 | 6220746 | NUDT1 | 650347 |
| ACOT7 | 4290050 | GNG11 | 1580025 | OAS2 | 7320561 |
| ACTN1 | 5080364 | GNLY | 5360064 | OLFM4 | 6770131 |
| ADA | 7210192 | GNLY | 6580041 | OLFML2B | 7150019 |
| ADM | 5670465 | GPNMB | 6840164 | OLIG1 | 2030403 |
| ADORA3 | 870138 | GPNMB | 1570154 | OLIG2 | 1780239 |
| AIF1 | 1940047 | GPR162 | 3520598 | OLR1 | 7510132 |
| AIF1 | 3800047 | GPR84 | 2070646 | ORM1 | 2850315 |
| ALAS2 | 4180768 | GRN | 1940021 | OSCAR | 1820450 |
| ALDH3B1 | 2970356 | GSN | 2370482 | PADI4 | 2650035 |
| ALDOA | 2640088 | GZMA | 3420612 | PAPLN | 5310364 |
| ALOX5 | 1780273 | GZMB | 1850523 | PARP9 | 3140707 |
| ALOX5AP | 3930343 | GZMH | 2370010 | PARP9 | 5700735 |
| ANKRD22 | 4150270 | H1F0 | 630278 | PDLIM7 | 4560164 |
| ANKRD22 | 130181 | H2AFY | 3930154 | PGCP | 2640377 |
| ANKRD57 | 2510224 | HAPLN3 | 4900086 | PGLYRP1 | 6250615 |
| ANPEP | 5810612 | HAS1 | 6270300 | PHLDA1 | 5900725 |
| AP1S2 | 1070192 | HBEGF | 1820594 | PI3 | 1050168 |
| APOBEC3G | 1190026 | HCK | 6270022 | PID1 | 1410114 |
| APOBEC3G | 4120369 | HIST1H1C | 5570279 | PLAUR | 6220671 |
| APOL3 | 1980750 | HIST1H2AC | 6590594 | PLAUR | 730528 |
| AQP9 | 160494 | HIST1H2BD | 290730 | PLAUR | 360475 |
| ARAP3 | 7380255 | HIST1H2BK | 6110630 | PLIN2 | 460204 |
| ARAP3 | 6040097 | HIST1H2BK | 5050402 | PLIN2 | 1400446 |
| ARMC10 | 2900372 | HIST1H4H | 2970019 | PLXDC2 | 5900497 |
| AVPI1 | 990500 | HIST2H2AA3 | 610451 | PMAIP1 | 2750367 |
| BASP1 | 2140121 | HIST2H2AA3 | 1820592 | PMP22 | 7560138 |
| BATF3 | 5900253 | HIST2H2AA4 | 4290148 | PNKD | 4730349 |
| BCAT1 | 7650524 | HIST2H2BE | 4230678 | PPAP2B | 7050575 |
| BCL6 | 6280458 | HK3 | 3060612 | PPARG | 830019 |
| BPI | 4200746 | HLA-DMA | 540563 | PPBP | 2230563 |
| BRI3 | 6270095 | HLA-DMB | 4900731 | PPP1R16B | 5490333 |
| C15ORF48 | 1110373 | HLA-DPA1 | 1190039 | PRF1 | 4670193 |
| C15ORF48 | 4070719 | HLA-DPB1 | 1050360 | PSAT1 | 4850674 |
| C17ORF60 | 4290056 | HLA-DQA1 | 780403 | PSMB8 | 3420632 |
| C19ORF59 | 1070367 | HLA-DRA | 2570564 | PSMB9 | 1940180 |
| C1ORF162 | 7050719 | HLA-DRA | 270168 | PSME2 | 830403 |
| C20ORF160 | 2900239 | HLA-DRB3 | 1770504 | PTGR1 | 2570332 |
| C5AR1 | 6450092 | HLA-DRB4 | 510079 | PTGR1 | 6450440 |
| **Gene Name** | **Probe ID** | **Gene Name** | **Probe ID** | **Gene Name** | **Probe ID** |
| CA12 | 3830075 | HLA-DRB4 | 7330398 | PTGS2 | 2260477 |
| CA12 | 150474 | HLA-DRB6 | 4200725 | PTGS2 | 1820632 |
| CAMP | 5860075 | HLA-DRB6 | 620544 | PYGL | 2760427 |
| CARD9 | 3710685 | HNMT | 3870102 | QPCT | 5570139 |
| CCL1 | 1030204 | HNMT | 4220168 | QSOX1 | 2850021 |
| CCL20 | 4220246 | HOPX | 3850246 | RAB31 | 6590341 |
| CCL3L1 | 6660288 | HPSE | 520162 | RAB32 | 2190128 |
| CCL8 | 6620121 | HPSE | 630619 | RAI14 | 6370187 |
| CCND2 | 3460520 | HS.407903 | 5720768 | RARRES3 | 2650564 |
| CCND2 | 1570348 | HSPE1 | 110110 | RETN | 4610129 |
| CD14 | 7000369 | HTRA1 | 730612 | RGS1 | 4490176 |
| CD14 | 6370369 | ICOS | 2070037 | RHOQ | 5050519 |
| CD163 | 2680092 | IDO1 | 5570711 | RHOU | 1230470 |
| CD163 | 20593 | IER3 | 1190367 | RIN2 | 6590445 |
| CD2 | 4210619 | IFI35 | 2510220 | RNASE1 | 4010296 |
| CD300C | 3120091 | IFI44 | 2570300 | RNASE2 | 2640068 |
| CD69 | 2710575 | IFI44L | 3870338 | RNASE3 | 3130543 |
| CD74 | 7040008 | IFIT2 | 2600747 | RNF130 | 1510722 |
| CD74 | 1240070 | IFIT3 | 1500280 | RNF144B | 70630 |
| CD74 | 4560047 | IFIT3 | 520408 | RPL14 | 3930008 |
| CD93 | 6100615 | IFITM1 | 5360156 | RRAS | 4570670 |
| CDA | 5090372 | IFNG | 630725 | RSAD2 | 3360343 |
| CDC45L | 2320170 | IFNGR2 | 2570291 | RXRA | 7000356 |
| CDCA7 | 2070520 | IL17F | 5360682 | S100A12 | 1410221 |
| CEACAM6 | 7400377 | IL18RAP | 6520180 | S100A4 | 5290270 |
| CEACAM8 | 4780075 | IL19 | 6330070 | S100A4 | 3990458 |
| CEBPB | 20446 | IL1A | 1980672 | S100A8 | 6060468 |
| CLEC1B | 6860164 | IL1F9 | 5890689 | S100A9 | 5390220 |
| CLEC4A | 2570022 | IL1RN | 2470601 | S100P | 1510424 |
| CLEC4A | 4050202 | IL1RN | 3060735 | SAMD9L | 3170136 |
| CLEC5A | 1260228 | IL24 | 1660746 | SDC2 | 2690026 |
| CSF1R | 1070014 | IL24 | 4290201 | SEPTIN5 | 6960022 |
| CSF2RA | 1980689 | IL24 | 6520347 | SERPINA1 | 730047 |
| CSF2RA | 4010719 | IL2RA | 130021 | SERPINA1 | 6380484 |
| CSF3R | 6270114 | IL2RB | 290402 | SERPING1 | 2030309 |
| CTLA4 | 6400333 | IL32 | 1400274 | SGK | 4390450 |
| CTLA4 | 1230201 | IL32 | 3440754 | SGK1 | 1410209 |
| CTSG | 1500735 | IL6 | 4040576 | SGK1 | 2030324 |
| CTSW | 6580408 | INDO | 380259 | SIGLEC10 | 4480497 |
| **Gene Name** | **Probe ID** | **Gene Name** | **Probe ID** | **Gene Name** | **Probe ID** |
| CXCL10 | 6270553 | IRF1 | 6250064 | SIGLEC14 | 1400593 |
| CXCL16 | 6280332 | IRG1 | 1770072 | SIRPA | 1940129 |
| CXCL2 | 4670390 | IRS2 | 6110736 | SIRPA | 5360193 |
| CXCL5 | 380010 | ISG15 | 2100196 | SLC11A1 | 780465 |
| CXCL5 | 540377 | ISG20 | 6330132 | SLC16A10 | 7550192 |
| CXCL9 | 5570278 | ITGAX | 4490500 | SLC16A3 | 110719 |
| CYB5R2 | 2940050 | ITGB5 | 2650114 | SLC16A6 | 5900564 |
| CYFIP1 | 4200343 | ITGB5 | 2490411 | SLC43A2 | 1820053 |
| CYP27A1 | 7040735 | KIAA1539 | 4880392 | SLC7A5 | 270152 |
| CYTL1 | 670731 | KIAA1598 | 5340162 | SLC7A7 | 4830632 |
| DAB2 | 610152 | KLRD1 | 1770598 | SLCO2B1 | 6580441 |
| DAB2 | 3930367 | KLRD1 | 3390594 | SLPI | 2140707 |
| DBNDD2 | 6420168 | KYNU | 3460685 | SMPDL3A | 4230669 |
| DEFA1 | 4540239 | KYNU | 7040142 | SNCA | 5360273 |
| DEFA1B | 7150170 | LAG3 | 2680189 | SOCS1 | 1990300 |
| DEFA1B | 4860128 | LAP3 | 3290292 | SORT1 | 160019 |
| DEFA1B | 870477 | LCN2 | 4390398 | SPI1 | 4220603 |
| DEFA3 | 2970747 | LILRA2 | 3370594 | SPI1 | 5810398 |
| DEFA4 | 6550164 | LOC100133583 | 3450736 | SPINK1 | 2260326 |
| DFNA5 | 580403 | LOC100133678 | 5310224 | ST3GAL6 | 5260440 |
| DLL1 | 4050687 | LOC389386 | 4780044 | STAB1 | 6040053 |
| DNER | 4570669 | LOC400759 | 620403 | STAT1 | 4810187 |
| DPYSL3 | 4290431 | LOC642567 | 5090382 | STAT1 | 2570079 |
| DUSP1 | 6860377 | LOC645638 | 70121 | STAT1 | 1820750 |
| ELANE | 7650497 | LOC653879 | 5560195 | TACSTD2 | 6270138 |
| EMILIN2 | 1190142 | LOC729009 | 2810463 | TAP1 | 7330392 |
| EMR2 | 2140136 | LOC730249 | 4670114 | TBXAS1 | 3940390 |
| EMR3 | 1030209 | LOC730415 | 7210253 | TCL1A | 70110 |
| EMR3 | 5270468 | LPL | 5560674 | TCN1 | 1770603 |
| ENG | 4610753 | LRRC25 | 5310397 | TFPI2 | 5080543 |
| EOMES | 7320372 | LTA | 1030743 | TGM3 | 4920075 |
| EPB41L3 | 1510538 | LTA4H | 1430524 | THBD | 5720369 |
| EPSTI1 | 5700725 | LY6E | 4890270 | THBS1 | 5810685 |
| FAM129B | 4070711 | LYN | 7320551 | TIMP1 | 7650433 |
| FAM129B | 3180053 | MAFB | 2060440 | TIMP2 | 780270 |
| FAM26F | 6590646 | MAP1LC3A | 5490240 | TMEM140 | 4670414 |
| FBP1 | 6020224 | MARCKS | 6060484 | TMEM158 | 3130220 |
| FCER1G | 3850440 | MCM10 | 7570181 | TNFAIP6 | 2140242 |
| FCGR2A | 450762 | MEGF9 | 1260136 | TNFRSF1B | 2490537 |
| **Gene Name** | **Probe ID** | **Gene Name** | **Probe ID** | **Gene Name** | **Probe ID** |
| FCGR2A | 1990278 | MERTK | 7550066 | TNFRSF4 | 650328 |
| FCGRT | 4200176 | MERTK | 4730315 | TNFSF10 | 870202 |
| FCN1 | 6660398 | METTL7B | 5310142 | TPST1 | 4760747 |
| FCRLB | 4730204 | MGST1 | 5080131 | TREM1 | 3310091 |
| FLJ10986 | 2650164 | MGST1 | 3180731 | TRIB1 | 4810520 |
| FLVCR2 | 1510026 | MIR155HG | 5860722 | TRIM22 | 5960747 |
| FOS | 4280017 | MMP1 | 3360224 | TYROBP | 2370358 |
| FPR1 | 1030270 | MMP10 | 150180 | UBD | 6420605 |
| FPR2 | 4150543 | MMP7 | 3800088 | UBE2L6 | 2070170 |
| FPR2 | 3400392 | MNDA | 6290270 | UBE2L6 | 7000368 |
| FTH1 | 5220240 | MS4A14 | 840689 | UHRF1 | 2940110 |
| FTHL11 | 460164 | MS4A14 | 5810170 | VAMP5 | 2630195 |
| FTHL3 | 6450139 | MS4A3 | 5050544 | VCAN | 5910113 |
| FTHL8 | 1980594 | MS4A7 | 990328 | VNN1 | 5310754 |
| GAGE12C | 2000673 | MS4A7 | 3840564 | VNN2 | 2690239 |
| GAGE12H | 3390039 | MT1F | 4220672 | VNN2 | 7100161 |
| GAGE2B | 3610544 | MT1G | 1170300 | VNN3 | 4010133 |
| GAPT | 6480079 | MT1H | 130093 | WARS | 4860224 |
| GAPT | 7570541 | MX1 | 1690066 | WARS | 3710068 |
| GBP1 | 2190148 | MXD1 | 670086 | WBP5 | 7510731 |
| GBP1 | 6840035 | NAMPT | 3060523 | XAF1 | 450189 |
| GBP2 | 1940162 | NAMPT | 2230379 | ZBED2 | 3940088 |
| GBP4 | 1980524 | NCF2 | 70008 | ZMIZ1 | 2450131 |
| GBP5 | 1510364 | NCRNA00085 | 2350156 | ZNF467 | 2490333 |
| GCA | 4230619 | NDFIP2 | 1030364 | ZSWIM4 | 1170072 |
| GCH1 | 240053 | NLRP12 | 520470 |  |  |
| GINS2 | 6020735 | NRP1 | 5720424 |  |  |

**Supplementary Table 8.** List of SDE genes when contrasting TB versus pneumonia for PPD stimulated samples.
